# *Plasmodium* gametocytes display homing and vascular transmigration in the host bone marrow

**DOI:** 10.1126/sciadv.aat3775

**Published:** 2018-05-23

**Authors:** Mariana De Niz, Elamaran Meibalan, Pedro Mejia, Siyuan Ma, Nicolas M. B. Brancucci, Carolina Agop-Nersesian, Rebecca Mandt, Priscilla Ngotho, Katie R. Hughes, Andrew P. Waters, Curtis Huttenhower, James R. Mitchell, Roberta Martinelli, Friedrich Frischknecht, Karl B. Seydel, Terrie Taylor, Danny Milner, Volker T. Heussler, Matthias Marti

**Affiliations:** 1 Department of Immunology and Infectious Diseases, Harvard T.H. Chan School of Public Health, 665 Huntington Avenue, Boston, MA 02115, USA.; 2 Institute of Cell Biology, University of Bern, Baltzerstrasse 4, CH-3012 Bern, Switzerland.; 3 Wellcome Trust Centre for Molecular Parasitology, Institute of Infection, Immunity and Inflammation, College of Medical Veterinary and Life Sciences, University of Glasgow, G12 8TA Scotland, UK.; 4 Department of Genetics and Complex Diseases, Harvard T.H. Chan School of Public Health, Boston, MA 02115, USA.; 5 Department of Biostatistics, Harvard T.H. Chan School of Public Health, Boston, MA 02115, USA.; 6 Department of Molecular and Cell Biology, Henry M. Goldman School of Dental Medicine, Boston University, Boston, MA 02118, USA.; 7 Broad Institute of Massachusetts Institute of Technology and Harvard, 415 Main Street, Cambridge, MA 02142, USA.; 8 Beth Israel Deaconess Medical Centre, Harvard Medical School, 330 Brookline Avenue, Boston, MA 02215, USA.; 9 Parasitology Centre for Infectious Diseases, University of Heidelberg Medical School, 69120 Heidelberg, Germany.; 10 Blantyre Malaria Project, University of Malawi College of Medicine, Blantyre 3, Malawi.; 11 College of Osteopathic Medicine, Michigan State University, East Lansing, MI 48824, USA.; 12 Department of Pathology, Brigham and Women’s Hospital, 75 Francis Street, Boston, MA 02115, USA.

## Abstract

Transmission of *Plasmodium* parasites to the mosquito requires the formation and development of gametocytes. Studies in infected humans have shown that only the most mature forms of *Plasmodium falciparum* gametocytes are present in circulation, whereas immature forms accumulate in the hematopoietic environment of the bone marrow. We used the rodent model *Plasmodium berghei* to study gametocyte behavior through time under physiological conditions. Intravital microscopy demonstrated preferential homing of early gametocyte forms across the intact vascular barrier of the bone marrow and the spleen early during infection and subsequent development in the extravascular environment. During the acute phase of infection, we observed vascular leakage resulting in further parasite accumulation in this environment. Mature gametocytes showed high deformability and were found entering and exiting the intact vascular barrier. We suggest that extravascular gametocyte localization and mobility are essential for gametocytogenesis and transmission of *Plasmodium* to the mosquito.

## Teaser

In vivo visualization of *Plasmodium* parasites reveals sublocalization, deformability, and mobility of gametocytes in the bone marrow.

## INTRODUCTION

Transmission to mosquitoes is an essential part of the *Plasmodium* parasite’s life cycle and a target of current intervention strategies. During transmission, gametocytes are ingested by mosquitoes where they undergo exflagellation and fertilization, leading to massive parasite replication. In the human-infective *Plasmodium falciparum* parasite, five developmental stages of gametocytes have been defined (referred to as stages I to V), of which only stage V forms are present in peripheral circulation (*1*). Postmortem analyses, as well as aspirates and biopsies of human tissues, have consistently revealed the presence of immature gametocytes (stages I to IV) in the bone marrow (BM) and the spleen of infected individuals (*2*–*7*). In a systematic analysis of human autopsies, we recently demonstrated that immature gametocytes are specifically enriched in the BM parenchyma, an extravascular niche that is separated from the blood by the vascular endothelium. In this niche, gametocytes are protected from immune clearance and are able to attach to and develop within erythroid precursor cells (*6*). These findings support a testable model in which parasites home to the BM, move across the endothelium, and upon maturation in the parenchyma intravasate into circulation as mature gametocytes. These processes require as yet uncharacterized modes of mobile behavior. Because such behavior cannot be tested in humans because of ethical reasons, we turned to the rodent malaria model *Plasmodium berghei*. Quantitative imaging experiments in this animal model and comparative histology in human autopsies provide first evidence for a conserved BM localization phenotype of *Plasmodium* gametocytes, involving selective tissue homing, transendothelial migration, and mobility of infected red blood cells (iRBCs).

## RESULTS

### *P. berghei* parasites accumulate in the extravascular niche of the mouse reticuloendothelial system

**Fig. 1:**
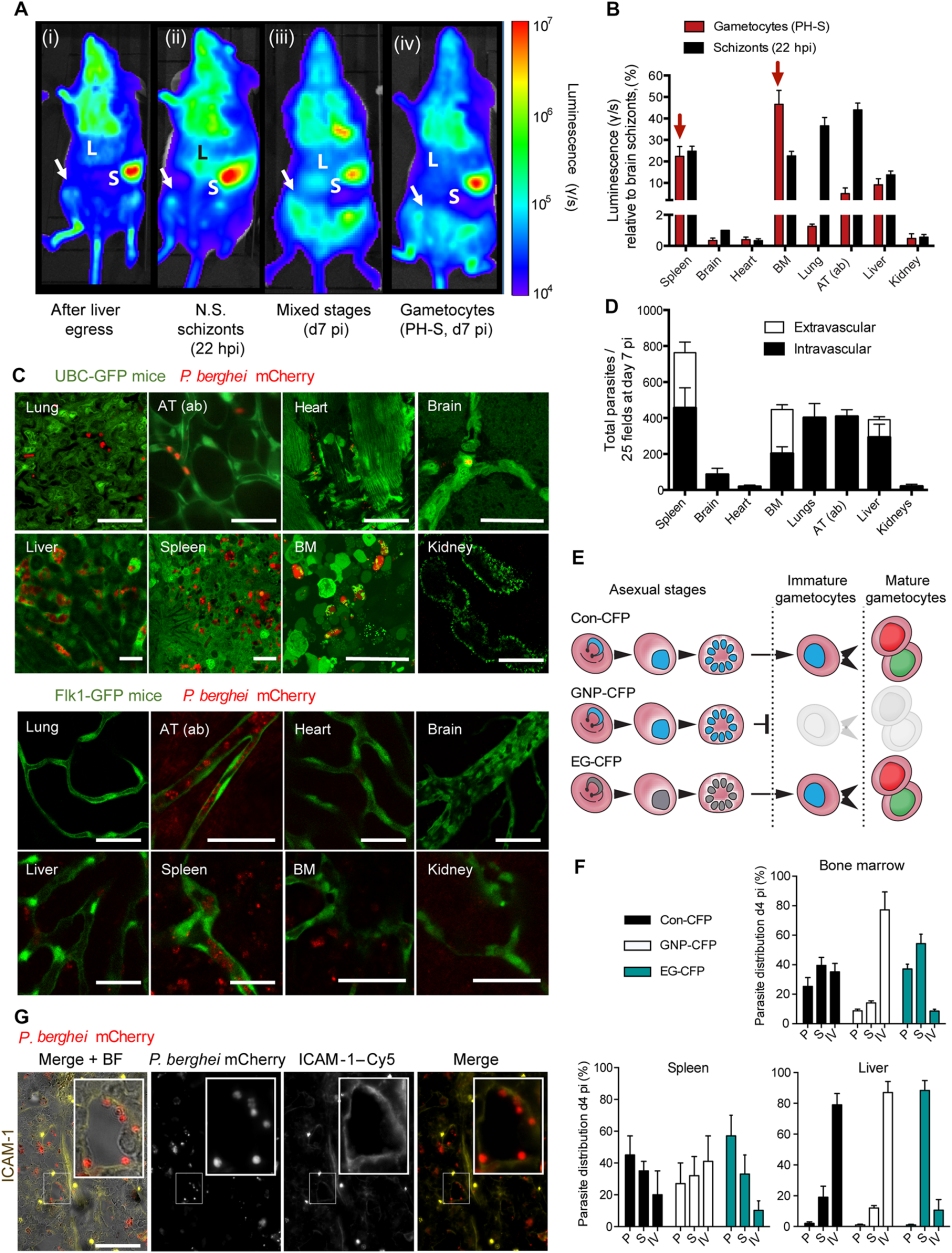
Parasite distribution in rodent tissues. (**A** to **C**) Bioluminescence and intravital imaging and ex vivo quantification in ice infected with transgenic *P. berghei* coexpressing mCherry and luciferase reporters (mCherry_Hsp70_-FLuc_ef1α_) (*36*). (A) Representative images show bioluminescence signal in the BM (femur and pelvic bones marked by arrow), in mice at (i) 65 hours following sporozoite infection, namely, after completion of the pre-erythrocytic stages, immediately following egress of merozoites from the liver to begin the first blood stage cycle; (ii) at 22 hours following infection with nonsequestering (N.S.) schizonts (that is, thereby, the only population detected at this time point are schizonts of the immediate next cycle) (*9*); and at day 7 (d7) postinfection (pi) with mixed blood stage parasites without (iii) or with (iv) pretreatment with PH-S (PH-S will select for gametocytes while killing the asexual populations of parasites). Note that the signal from the BM is potentially obscured by that of the adipose tissue in (iii), but not in (ii) because of the lack of sequestration phenotype. hpi, hours post-invasion. (B) Bioluminescence-based quantification of individual extracted organs from 15 mice (5 in triplicate experiments) infected with either synchronous schizonts (black bars) or after PH-S treatment (red bars). Bars indicate luminescence (γ/s), normalized to and expressed as a ratio of schizont signal in the brain. Arrows show that the largest relative gametocyte signal occurs in spleen and BM. AT (ab), abdominal adipose tissue. (C) Representative intravital and/or ex vivo images of mCherry_Hsp70_-FLuc_ef1α_ *P. berghei* gametocytes (red) in various tissues of transgenic mice treated with PH-S (green) expressing either UBC-GFP (top panels) or Flk1-GFP (bottom panels). Lung, abdominal adipose tissue, heart, and brain show gametocytes in vasculature, whereas liver, spleen, and BM also show extravascular gametocytes. Scale bars, 20 μm. (**D**) CD31- and Flk1-based immunofluorescence quantification of vascular versus extravascular mCherry_Hsp70_-FLuc_ef1α_ *P. berghei* distribution in mice after asexual blood stage infection. Highest extravascular parasite levels are observed in the spleen, BM, and liver (white stacked bars). Error bars represent SDs of quantifications in 25 fields of view across 18 separate mice. (**E**) Schematic showing specific fluorescence of gametocyte producer (Con and EG) or nonproducer (GNP) *P. berghei* lines, under constitutive (Con-CFP and GNP-CFP) or early gametocyte–specific promoters (EG-CFP). (**F**) Distribution of gametocyte producer and nonproducer lines within the parenchyma (P), sinusoids (S), and intravascular (IV) spaces of BM, spleen, and liver of mice at day 4 pi. Con-CFP parasites are equally distributed across BM and spleen compartments. In contrast, EG-CFP parasites are enriched in parenchyma and sinusoids, whereas GNP parasites are enriched in the vasculature. Error bars represent SDs of quantification in 25 fields of view across 30 separate mice. (**G**) Extravascular parasites in the BM localize to sinusoids and the parenchyma, as defined by ICAM-1 labeling (yellow). The inset in the ICAM-1 image shows gametocytes in close distance to the sinusoidal lining (left panel). Scale bars, 20 μm. For all experiments, three to five mice were used per triplicate experiment, and at least 25 fields of view were quantified per mouse.

The presence of *P. berghei* parasites across several mouse tissues was assessed by bioluminescence and fluorescence imaging methods. To specifically investigate gametocyte tissue distribution, we induced gametocytogenesis and eliminated asexual parasite populations using a combined treatment of phenylhydrazine and sulfadiazine (PH-S) (as described in Materials and Methods) (*8*). In addition, we used transgenic parasite lines with fluorescent reporters to differentiate asexual from gametocyte stages (note that all parasites analyzed except for invasive merozoites are contained within an RBC). We observed gametocyte enrichment in the reticuloendothelial system (BM, spleen, and liver) of Balb/c and C57BL/6 mice at multiple times after infection. Comparative intravital, ex vivo, and bioluminescence imaging revealed an enrichment of both schizonts and gametocytes in BM, liver, and spleen (Fig. 1, A and C, and fig. S1A), although schizonts preferentially accumulated in the lungs and adipose tissue, as described previously (*9*, *10*). Analysis of transgenic mice expressing green fluorescent protein (GFP) under the control of the ubiquitin promoter in all tissues (UBC-GFP) (*11*), or expressing GFP in the vascular endothelium across all tissues (Flk1-GFP) (*12*), further demonstrated significant extravascular parasite enrichment in BM, spleen, and liver (Fig. 1, C and D). This in vivo evidence for parasite endothelial localization was confirmed by ex vivo staining with antibodies specific for CD31 and ICAM-1 (intercellular adhesion molecule–1), markers for the vascular endothelium (fig. S1, B to D). These results confirm early observations by Weiss (*13*) and Singer (*14*) who first described parasites in this location in the rodent model. Next, we used a series of transgenic parasite lines with stage-specific fluorescent reporters (*15*) including constitutive cyan fluorescent protein (Con-CFP), early-gametocyte CFP (EG-CFP), and gametocyte nonproducer CFP (GNP-CFP) lines to directly differentiate asexual from gametocyte stages and define their localization across tissues by quantification in ex vivo tissue sections (Fig. 1E; see also fig. S1E for methods). Gametocytes were highly enriched in the parenchyma and sinusoids of BM and spleen and in liver sinusoids, whereas most asexual parasites were found in the peripheral vasculature of most organs (Fig. 1, F and G). Overall, the localization of parasites across mouse tissues and the preferential localization of gametocytes to the extravascular niche of the reticuloendothelial system (that is, sinusoids and parenchyma) recapitulated our previous findings in human autopsies (*6*) and recent data from the humanized mouse model (*16*). Notably, extravascular parasites in mice are largely protected from phagocytosis by macrophages (fig. S2, A and B), consistent with previous observations in human autopsies (*6*).

### Selective homing of a subset of parasites to the BM niche

**Fig. 2:**
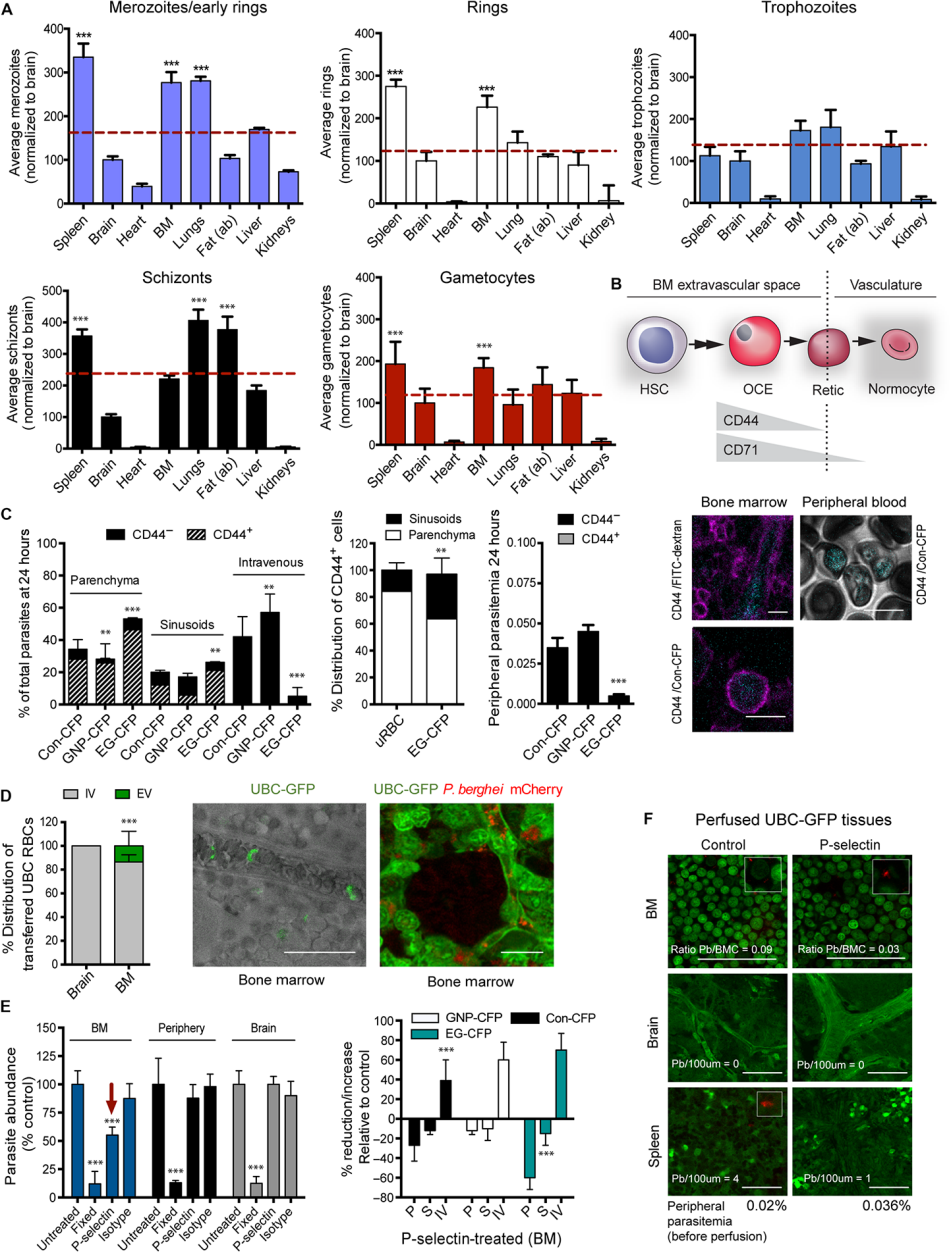
Parasite homing and extravascular BM localization during mouse infection. (**A**) Synchronous infections of mice with mCherry_Hsp70_-FLuc_ef1α_ merozoites, rings, trophozoites, schizonts, or gametocytes. Data show that merozoites, rings, and gametocytes are significantly enriched in BM and spleen (red arrows, ^***^P < 0.001) compared to mean across all organs (dashed line). Data are expressed relative to parasite numbers quantified in the brain (normalized to 100) and correspond to intravascular and extravascular compartments in all organs. (**B**) Representative diagram of hematopoietic development. Hematopoietic stem cells (HSCs) undergo a series of developmental steps until formation of the final nucleated stage termed orthochromatic erythroblast (OCE), which upon enucleation gives rise to reticulocytes (Retic). Reticulocytes intravasate into circulation and rapidly mature to terminal normocytes. Extravascular RBC precursor cells are marked by CD44, whereas CD71 is lost shortly after intravastion. (**C**) Presence of stage-specific reporter parasites in CD44^+^ RBCs following injection of merozoites. At 24 hpi, EG-CFP parasites, but not GNP-CFP and Con-CFP parasites, are largely confined to parenchyma and sinusoids where most are present in CD44^+^ RBCs (left panel), reflecting distribution of CD44^+^ RBCs (mid left panel). No parasites are detected in CD44^+^ cells in peripheral blood (mid right panel). Representative images of CD44^+^ cells in a BM section (magenta) are shown, upon injection of fluorescein isothiocyanate (FITC)–dextran (cyan) to mark vasculature (top panel). Bottom image: Close-up of an infected CD44^+^ RBC in BM. (**D**) Presence of uninfected UBC-GFP RBCs in the BM extravascular niche. UBC-GFP RBCs were obtained from transgenic donor mice and injected into naïve, uninfected recipient mice and quantified in BM. A small proportion of RBCs accumulates in BM extravasculature but not in the brain extravascular niche (left panel). Representative image is shown (left) and a control with mCherry_Hsp70_-FLuc_ef1α_ *P. berghei* parasites (right). (**E**) Inhibition of parasite extravasation using receptor antibodies. Mice were pretreated for 24 to 48 hours with P-selectin antibodies before injection of mCherry_Hsp70_-FLuc_ef1α_ merozoites and compared to untreated and nonviable (4% paraformaldehyde-fixed) control parasites (left panel). Parasite abundance in BM but not in brain or peripheral circulation is reduced upon P-selectin pretreatment compared to controls. Injection of merozoites from stage-specific reporter parasites upon P-selectin pretreatment reveals greatest reduction in BM parenchyma with EG-CFP and Con-CFP parasites, whereas intravascular localization is increased (right panel). Error bars represent SEs from five separate experiments in three mice per condition. (**F**) Quantification of peripheral parasitemia upon treatment with receptor antibodies. UBC-GFP mice were pretreated for 24 to 48 hours with P-selectin before injection of mCherry_Hsp70_-FLuc_ef1α_ merozoites (right panel) and compared to untreated control parasites (left panel). Peripheral parasitemia and tissue burden were quantified before and after cardiac perfusion to remove circulating parasites—leaving only those in the extravascular spaces. Extravascular parasite load decreases significantly in BM and spleen compared to control animals, whereas peripheral parasitemia (obtained before perfusion) increases. *P* values are shown as **P < 0.05 and ***P < 0.005. All error bars represent SDs of the mean.

**Fig. 3:**
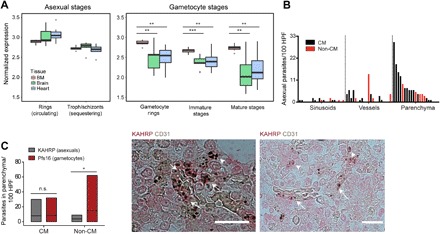
Asexual and gametocyte stages in the BM parenchyma of human malaria cases. (**A**) NanoString analysis of BM, brain, and heart tissue from patients who died of clinical cerebral malaria. Asexual *P. falciparum* parasite stages are predominantly sequestered in the brain and heart (left panel). All gametocyte-specific transcripts are significantly enriched in BM compared to the other tissues (right panel). Data were normalized on the basis of background subtraction and expression of housekeeping genes. **P < 0.01 and ***P < 0.001. (**B** and **C**) Quantification of immunohistochemistry (IHC) studies on human autopsy cases from BM tissue (*n* = 22). (B) Quantification of IHC with KAHRP antibodies to mark asexual *P. falciparum* parasites and CD31 to mark vasculature (HPF, high-powered fields). Histogram demonstrated that presence of parasites in BM is highest in the parenchyma compared to sinusoids and vessels. (C) Patients succumbing in absence of cerebral malaria (CM) had higher numbers of gametocytes (Pfs16) in parenchyma than asexual parasites (KAHRP), whereas these numbers were similar in CM patients. Representative IHC images of CD31 (brown) and KAHRP (red) from a human autopsy BM sample are shown. Arrows indicate intravascular asexual parasites, and arrow heads indicate extravascular asexual parasites. *P < 0.05. Scale bars, 20 μm.

Two mutually compatible models have been proposed to explain gametocyte enrichment in the extravascular space of the BM in humans (*17*): (i) selective homing of a subset of parasites [either sexual merozoites (that is, merozoites contained in a sexually committed schizont) or young gametocytes] to BM from an outside site of formation or (ii) an endogenous asexual cycle promoting replication and gametocyte formation in the BM. To test these models, *P. berghei* parasites were synchronized ex vivo and injected either directly into the recipient mice or to intermediate donor mice for further development. Synchronized infections of individual parasite stages demonstrated that parasites injected directly after mechanical rupture of schizonts to release merozoites, as well as purified ring stages and purified gametocytes, showed highest enrichment in BM, spleen, and lungs within 2 hours after infection (Fig. 2A). In each case, stages were synchronized before injection, and each of the stages was specific (that is, no mixed stages were injected). To determine whether merozoites invade RBCs in the BM or en route, we quantified the fraction of injected merozoites that was present in BM-resident RBC precursor cells (determined by CD44^+^ expression) (Fig. 2, B and C) (*18*). At 24 hours after infection, most of the gametocytes were present in RBC precursor cells in the BM parenchyma and sinusoids, whereas asexual parasites were equally distributed in BM extravascular and intravascular compartments and in the peripheral circulation at any time after infection (Fig. 2C). This localization pattern was consistent between 2 hours and 4 days after infection (fig. S2C). Together, these observations support both models: They strongly suggest that asexual and gametocyte stages can form in the BM upon merozoite invasion of BM-resident RBCs; the presence of parasites in CD44^−^ RBCs and efficient BM homing of ring stages (Fig. 2A) suggests that circulating iRBCs may also enter the extravascular niche, similar to observations made with uninfected RBCs (uRBCs) (Fig. 2D). The presence of a subset of parasites in CD44^−^ RBCs may also be the result of accelerated RBC maturation upon parasite infection, as observed in *Plasmodium vivax* (*19*). To determine whether parasite localization in the extravascular niche depends on specific parasite-host interactions, we blocked endothelial receptors using antibodies against P-selectin, E-selectin, ICAM-1, and VCAM-1 (vascular cell adhesion molecule–1) or a control antibody before merozoite infection into mice. Parasite burden in BM was significantly decreased when specific receptors were blocked individually or in combination, whereas circulating parasite levels remained unaffected (Fig. 2, E and F, and fig. S2D). In particular, gametocyte localization in the BM parenchyma was reduced by 60% compared to control when host P-selectin was blocked. Together with the predominant presence of gametocytes in CD44^+^ cells in the BM parenchyma (Fig. 2C), these data may support a mechanistic model where a subset of merozoites extravasates across the sinusoidal endothelium via specific receptor-ligand interactions, reminiscent of homing and vascular transmigration of immune cells (*20*). Increased levels of parasite accumulation in BM including extravascular parasites in CD44^−^ cells were detected later during infection (figs. S2C and S3A), coinciding with the emergence of vascular leakage in BM and spleen as a result of inflammation (fig. S3B and movie S1). Inspection of BM tissue from *P. falciparum*–infected patients with fatal cerebral malaria revealed the presence of significant levels of asexual parasites and gametocytes in the BM parenchyma (Fig. 3, A to C). Together, these observations suggest that acute infection facilitates further parasite infiltration of the extravascular niche, including replicative asexual stages, through vascular leakage.

### Direct evidence for parasite translocation across the BM endothelial barrier

**Fig. 4:**
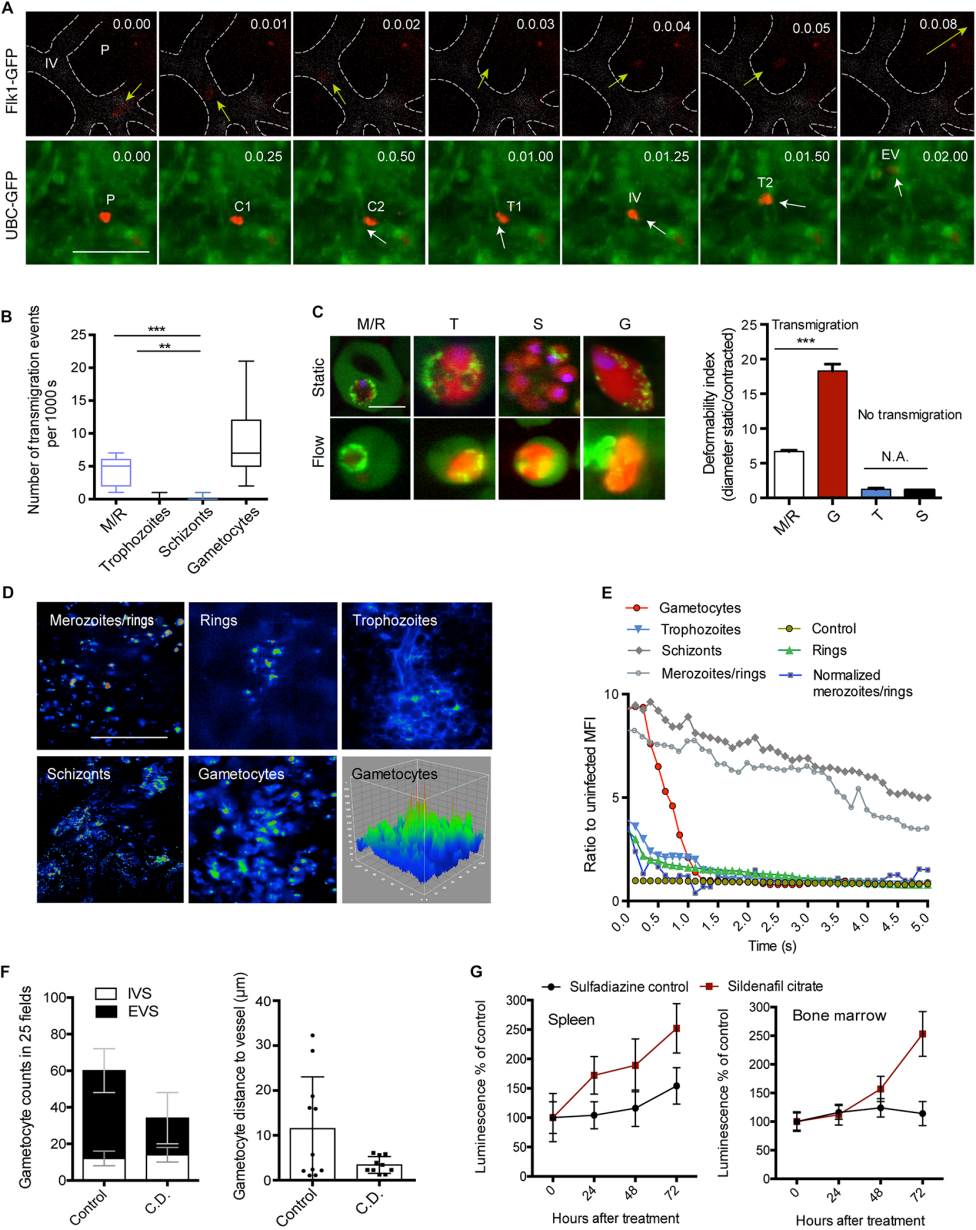
Gametocyte transmigration dynamics and vascular leakage. (**A**) Time series of mCherry_Hsp70_-FLuc_ef1α_ gametocyte crossing the vascular endothelium in an Flk1-GFP (top panel) or UBC-GFP (bottom panel) transgenic mouse. [Time lapse, 5 frames/s; scale bar, 10 μm.] P, parasite; IV, intravascular; EV, extravascular; C1,2, contact site; T1,2, transmigration. In the top panel series, a gametocyte is exiting the BM vasculature (marked as dotted lines), whereas the bottom panel series are showing a gametocyte entering the BM vasculature. (**B**) Quantification of observed transmigration events across the BM vascular barrier. Upon injection of synchronized parasite stages, only merozoites and gametocytes show significant levels of transmigration events. (**C**) Sphericity and deformability of *P. berghei* parasites. Left: Representative in vivo images across stages of mCherry_Hsp70_-FLuc_ef1α_ parasites within UBC-GFP RBCs in static and flow conditions (scale bar, 5 μm). Right: Deformability indices across stages defined as the ratio of maximum cellular diameter before (static) and during (contracted) transmigration (*n* = 100; bars represent mean, and error bars represent SD. ***P < 0.001). (**D** and **E**) Transmigration and vascular leakage. Mice are injected with synchronous mCherry_Hsp70_-FLuc_ef1α_ parasite stages followed by FITC-dextran inoculation, and images are taken at a rate of 5 fps. Representative images of FITC-dextran fluorescence intensity are shown (D). Quantification of FITC-dextran leakage (representative of peripheral blood including iRBCs and uRBCs), demonstrating a unique transient leakage pattern upon injection of mature gametocytes (E). Note that merozoites/rings (M/R) values are normalized by schizont values because of the observed low level of sustained leakage pattern likely caused by co-injected hemozoin. MFI, mean fluorescence intensity. (**F**) Gametocyte treatment with Cytochalasin D (C.D.) abrogates extravascular accumulation. mCherry_Hsp70_-FLuc_ef1α_ gametocytes are purified ex vivo and incubated with C.D. (or vehicle control) before washout and intravenous injection into naïve mice. Relative abundance and distance from vessels in the extravascular BM compartment are significantly reduced. IVS, intravascular space; EVS, extravascular space. (**G**) Treatment of infected mice with sildenafil citrate results in gametocyte accumulation in BM and spleen. Mice treated with phenylhydrazine were infected with mCherry_Hsp70_-FLuc_ef1α_ parasites and treated with sulfadiazine–sildenafil citrate for 3 days (or sulfadiazine only, control). Treatment leads to significantly increased accumulation of gametocytes in the BM and spleen of mice compared to control. In all panels, error bars represent SDs of the mean. *** *P* < 0.001 and **P < 0.01. N.A., not applicable.

Preferential accumulation of gametocytes in the extravascular niche, including the parenchyma, suggests that mature gametocytes are capable of crossing the endothelial barrier. Using a combination of transgenic mice expressing endothelial markers and vascular staining revealed multiple mature gametocyte translocation events (Fig. 4A and movies S2 and S3) across the sinusoidal barrier of BM, spleen, and liver. Quantification of individual translocation events in mice injected with equal amounts of synchronized parasite stages revealed substantial crossing events for gametocytes, whereas no events were detected for trophozoites and schizonts (Fig. 4B). Characterization of transmigration events showed significantly altered cellular diameter [that is, deformation index (*21*)] of mature gametocytes during transmigration, whereas young parasites transmigrated with little measurable deformation (Fig. 4C). This measure is approximate and limited by the resolution possible using intravital microscopy (IVM). Taking this technical limitation into account, gametocytes showed a deformation index of up to 20-fold upon their passage through vessels both in the spleen and the BM compared to their uncompressed size. Gametocytes also displayed a unique dextran permeability pattern during endothelial transmigration compared to the other stages (Fig. 4, D and E, fig. S4, and movie S2), suggesting a mechanism of passage similar to highly motile and deformable immune cells such as leukocytes during acute inflammation. Endothelial transmigration of leukocytes requires actin-dependent motility that can be inhibited with cytochalasin D (*22*), a potent inhibitor of actin polymerization. Previous work demonstrated that short-term treatment of *P. falciparum* with cytochalasin D at 1 μM depletes F-actin at the tip of the polarized gametocyte (*23*) and inhibits the actin-dependent process of merozoite invasion (*24*) without affecting viability. Here, we showed that similar pretreatment of *P. berghei* gametocytes with cytochalasin D before injection into Flk1-GFP reporter mice resulted in >50% reduced extravascular gametocyte numbers. Furthermore, gametocytes present in the extravascular space were located closer to vessels compared to untreated parasites (Fig. 4F), perhaps suggesting impaired mobility. Treatment of infected mice over 3 days with sildenafil citrate, a phosphodiesterase inhibitor known to keep *P. falciparum* gametocytes in a rigid state (*25*), led to increased bioluminescent signal in the BM and spleens of infected mice (Fig. 4G). These data support a model where sildenafil citrate impedes the capacity of mature gametocytes to transmigrate; however, further work is required to fully characterize this phenotype. Together, these observations provide initial mechanistic insight and suggest that *P. berghei* gametocytes exhibit some as yet uncharacterized mode of actin-dependent deformability and/or mobility.

### *P. berghei* gametocytes exhibit mobile behavior in the BM

**Fig. 5:**
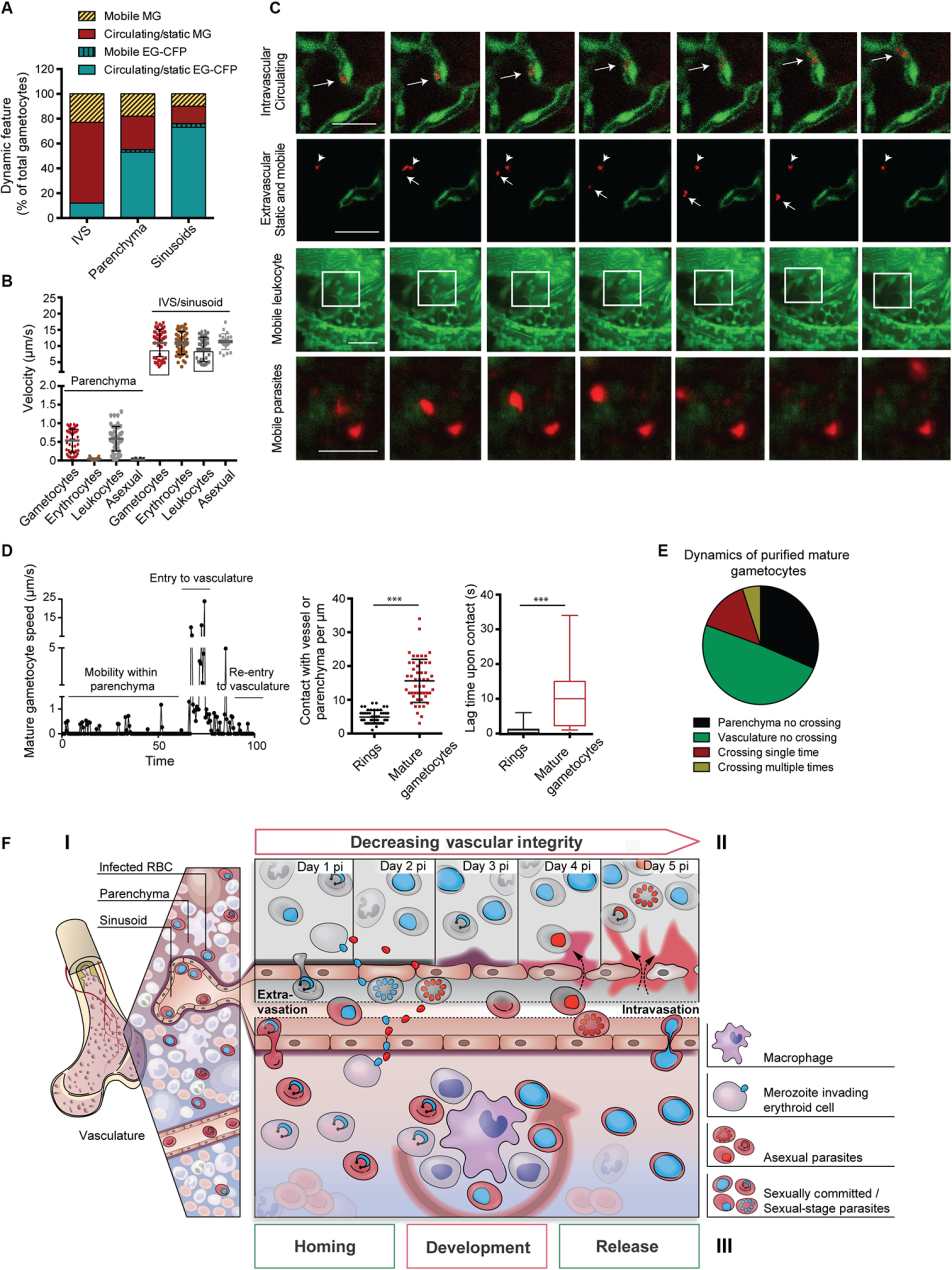
Gametocytes display mobility behavior in tissues of the reticuloendothelial system. (**A**) Gametocytes are mobile in the BM and spleen. Early (EG) and mature (MG) gametocyte behavior is quantified across intravascular and extravascular compartments. Most mobile gametocytes are mature; most of the mature gametocytes are found passively circulating in the vasculature, whereas a subset is present in all compartments. Early gametocytes are confined to the extravascular niche and static. (**B**) Mature gametocytes move at speeds between 0.01 and 1 μm/s in the parenchyma of BM, similar to leukocytes. A subset of gametocytes and leukocytes (black box) show contact-dependent movements in the sinusoids and small vessels of the same organs at a speed between 4 and 10 μm/s. In contrast, uninfected erythrocytes and asexual parasite stages are static in the parenchyma and passively move with the flow in sinusoids and small vessels at a much higher speed. (**C** and **D**) Time-lapse intravital images of gametocyte behavior in the BM or spleen. *P. berghei* mCherry gametocytes are imaged in Flk1-GFP mice. Top panel: Mature gametocyte circulating within the vasculature (arrow). Second panel: Mobile (arrow) and static (arrow head) gametocytes in the BM extravascular space of Flk1-GFP mice. Third panel: Motile behavior of leukocytes in uninfected UBC-GFP mice. Bottom panel: Mobile behavior of gametocytes in infected UBC-GFP mice. (D) Mobility pattern of one gametocyte dynamically moving in the BM. Mature gametocytes show multiple mobility patterns in the spleen and BM: multiple contact points in parenchyma (represented by 0 in the *y* axis) followed by forward motion (left panel) and rapid translocation upon contact with sinusoidal endothelium. Compared to ring stages, gametocytes contact the parenchyma two to four times more frequently (middle panel). At every contact point leading to a stop phase, gametocytes display a lag in velocity of up to 35 s (right panel). (Graphs show median and interquartile ranges of lag time following contact.) Error bars show SEM. ***P < 0.001. (**E**) Mature gametocytes display various behaviors in relation to crossing the vascular endothelium. Most of the parasites recorded were in the vasculature and not crossing, whereas a subset is either crossing once or twice. (**F**) Hypothesized model of parasite dynamics in the BM. (I) Distribution of *Plasmodium* gametocyte stages in the BM. Early gametocyte stages accumulate in the parenchyma and sinusoids of BM (and spleen), whereas mature gametocytes are found both intra- and extravascularly. (II) Vascular leakage and its relation to asexual parasite accumulation in BM. Disease progression coincides with vascular leakage in sinusoids of the reticuloendothelial system but not in the vasculature of other tissues. This leakage results in increased accumulation and replication of asexual parasite stages in the BM parenchyma. (III) Mobility and vascular migration in the BM. A subset of merozoites, including most of the sexual merozoites, invades BM-resident RBC precursor cells in sinusoids and parenchyma. The latter requires merozoite extravasation across the sinusoidal barrier via specific receptor-ligand interactions. Early gametocytes are located on the sinusoidal lining and attached to erythroblastic islands in the parenchyma, where they develop until maturity (represented by red arrow). Mature gametocytes become mobile, presumably coinciding with a switch in deformability, and enter circulation from their extravascular niche.

Next, we systematically analyzed the behavior of different developmental gametocyte stages by intravital imaging. Monitoring gametocyte maturation and in vivo behavior upon PH-S treatment of infected mice revealed the presence of immature gametocytes in BM, spleen, and liver (fig. S5A), whereas mature stages were detectable across all tissues. As suggested by the gametocytes’ proximity to blood vessels after cytochalasin D treatment (Fig. 4F), we observed that a significant fraction of mature gametocytes were mobile within the BM and spleen, both in the intravascular and extravascular spaces. Others remained static in the extravascular space or were circulating at the same speed as RBCs in the intravascular space [Fig. 5, A to D, fig. S5B, movies S2 and S4 (mobile), and movies S5 to S12 (circulating/static)]. Parenchymal velocity of mobile gametocytes ranged between 0.01 and 1.0 μm/s, whereas no mobility was observed for uRBCs and those infected with asexual parasites. In the sinusoids and small vessels, a fraction of mature gametocytes was observed at speeds of 5 to 10 μm/s (Fig. 5B). These values are very similar to the velocity reported for rolling leukocytes in these compartments (Fig. 5, B and C) (*20*). Overall, we defined three key characteristics of mature gametocyte mobility: (i) it often requires contact with other cells (Fig. 5, C and D), (ii) the lag time at each contact point is more extended than that of asexual ring stage controls and reminiscent of rolling leukocyte movement (Fig. 5D, right panel), and (iii) transmigration of mature gametocytes across vessels is not a rare event and individual gametocytes can transmigrate more than once (Fig. 5E). Suggestions of gametocyte mobility in vivo have never been reported; however, recent studies on *P. falciparum* gametocyte cellular structure have defined an architecture potentially compatible with motile “zoite” forms of the parasite such as sporozoites and ookinetes, although gametocytes might use a different mode of movement, or the movement observed might be passive (*23*, *26*). Both the patterns of mobility and the contact-dependent movement in parenchyma and vessel walls that we observed in our work have close similarities with the process of rolling, adhesion, and transmigration characteristic of immune cell types [reviewed in the studies of Vestweber (*27*), Nourshargh *et al*. (*28*), Fackler and Grosse (*29*)]. Intravital images of leukocyte motility are included in movie S13. Despite some similarities captured by microscopy, targeted mechanistic studies are required to determine whether the observed mobility is intrinsic to the gametocyte, whether it is a result of overall movement of BM-resident cell populations through time, or whether it represents peristaltic and heartbeat-induced movements (or a mixture of all the above phenomena). Nevertheless, other *P. berghei* stages, namely trophozoites, schizonts, and ring stages, do not display this type of movement.

## DISCUSSION

*P. falciparum* gametocytes were first detected by Auguste Laveran in the 1880s; however, major knowledge gaps in their biology still exist. Marchiafava and Bignami (*2*) initially detected gametocytes in autopsy material and hypothesized their sequestration in tissue niches. Additional historical case studies of malaria patients have indicated presence of the human malaria parasite *P. falciparum* in BM and spleen (*3*, *4*). Our systematic investigation in a cohort of fatal pediatric malaria cases has demonstrated significant enrichment of transmission stages in the BM extravascular niche (*6*). Parallel comparative investigation of BM aspirates and peripheral blood samples from anemic children revealed immature gametocyte enrichment in the BM niche (*7*). Finally, recent data in humanized mice demonstrated some gametocyte enrichment in BM (*16*). The biological relevance of these observations remained unclear and represents one of the key knowledge gaps in the parasite cycle. Here, we have used a series of complementary approaches to demonstrate extravascular parasite development in the rodent malaria parasite *P. berghei*. In contrast to human biopsy cases and the highly artificial humanized mouse model, the establishment of a solid rodent model to study the dynamics of gametocyte homing to the different tissues depending on its developmental stage opens completely new roads for the research on sexual *Plasmodium* stages.

We demonstrate here that preferential gametocyte development in the BM and spleen extravascular niche is present in the rodent malaria model, suggesting conservation of this phenotype across *Plasmodium* lineages. We provide conclusive evidence for the selective and receptor-mediated homing of a subset of merozoites to the BM where they initiate extravascular parasite development upon invasion of RBC precursor cells. These observations suggest the existence of distinct merozoite populations: sexual merozoites with restriction (or at least strong preference) for BM and spleen homing and extravasation and asexual merozoites with no such tissue restriction. We hypothesize that sexual merozoites display a set of unique surface ligands that provide the selectivity for interactions with BM and spleen endothelial receptors such as P-selectin, in addition to those required for RBC invasion. We also detected asexual parasite stages in the extravascular environment both in *P. falciparum* (in human autopsies) and in *P. berghei* (in infected mice), suggesting the existence of a genuine extravascular asexual cycle in both *Plasmodium* lineages. If confirmed, then such an extravascular replication cycle may represent a parasite reservoir independent of the major asexual parasite population in the vasculature, with possible implications for diagnosis and treatment.

Intravital imaging experiments in infected mice provide direct evidence for transmigration across the endothelial barrier and yet uncharacterized modes of mature gametocyte mobility in the extravascular and intravascular environments of BM and spleen, both characteristics of immune cells such as leukocytes. Specifically, we observed highly deformable mature gametocytes crossing the endothelial barrier in both directions. A series of studies in *P. falciparum* have demonstrated that immature gametocytes are rigid before switching to a deformable state during final maturation (*26*, *30*, *31*). This deformability switch involves a adenosine 3′,5′-monophosphate–dependent signaling cascade that can be blocked with specific inhibitors such as sildenafil citrate (*25*). Here, we demonstrate that sildenafil citrate accumulates gametocytes in BM (and spleen), suggesting that the switch to a deformable state is required for the intravasation of mature gametocytes into circulation (and subsequent passage through the spleen). Further experiments are required to rigorously test this hypothesis in the animal model and under controlled in vitro conditions. We also observed that mature gametocytes were changing location in the parenchyma at speeds similar to leukocytes (~0.5 μm/s), whereas asexual parasites and uRBCs remained static. Similarly, a subset of mature gametocytes displayed contact-dependent movements in sinusoids and vasculature similar to leukocytes (5 μm/s). The observed restriction of these phenotypes to BM and spleen suggests that a specific microenvironment is required to induce gametocyte mobility. Intriguingly, our experiments also revealed that mature gametocyte homing to BM and their extravasation are inhibited by the actin-depolymerizing agent cytochalasin D. Together, our data suggest that mature gametocyte mobility displays many hallmarks of immune cells. Given that gametocytes are intracellular, they must provide machinery to a host cell that otherwise is incapable of the observed phenotypes. Notably, RBC precursor cells reach the endothelial barrier and intravasate during their maturation. Our data demonstrate that gametocyte development is largely restricted to these RBC precursors because of their predominance in the extravascular environment, and therefore, gametocytes may modify their young host cell to ensure that it retains these properties.

Our hypothesized model for gametocyte dynamics based on the findings of this work is summarized in Fig. 5F. These findings define the BM as a key compartment in the *Plasmodium* life cycle, with major implications for our understanding of parasite biology, diagnosis, and the development of novel transmission-blocking interventions.

## MATERIALS AND METHODS

### Study design

Given our previous evidence in human autopsy case studies of enrichment of *Plasmodium* parasites in the BM and spleen (*6*), we aimed to investigate the distribution of asexual parasites in BM tissues using stage-specific antibodies. For this purpose, we analyzed the same subset of autopsy samples that were used for the initial investigation of BM enrichment in our original study.

In a second line of investigation, we aimed to define presence and dynamics of parasites in rodent models. To do this, we established IVM methods and rigorously quantified parasites by fluorescence microscopy in vivo and ex vivo in all organs as stated. Each experiment was performed at least in triplicate, with five mice per repeat, per group and/or per condition. We performed power analyses to calculate sample sizes. In specific experiments where the number of events in 15 mice was not sufficient to reach this power, we increased the number of mice used. The rule that we adopted to stop data collection was the development of cerebral malaria symptoms in C57BL/6, UBC-GFP C57BL/6, or FLK-GFP C57BL/6 mice; however, most of these mice were not used to measure parasite loads at the time window or parasitemia numbers where experimental cerebral malaria is expected. We did not exclude any outlier. The research subjects in all cases were either C57BL/6 mice [wild-type (WT) or fluorescent reporter mice] or Balb/c mice. *P. berghei* ANKA parasites were used throughout the study. Treatments applied to the animals included infections, drug treatments, dye injections to visualize the vasculature, and antibody treatments. The outcomes of infection were quantified by luminescence or fluorescence in vivo by IVM or bioluminescence imaging, or ex vivo following organ extraction, using confocal microscopy or bioluminescence imaging. Animals were randomly divided into cages of the different experimental groups, and animals of the same age and batch (often siblings) were selected each time for the various assays. Blinding was performed at stages where this was possible; however, not all treatments or interventions were blinded.

### Patient samples and ethical approval

Patient samples used in this study were from autopsies performed as part of the pediatric severe malaria postmortem study conducted between 1996 and 2011, and the patient demographic information and sample collection procedure were previously described (*6*). The autopsy cohort study was approved by the institutional review boards at the University of Malawi College of Medicine, Michigan State University, and Harvard T.H. Chan School of Public Health. Informed written consent was obtained from all parents/guardians of the enrolled patients. Criteria used for diagnosis and clinical management have been previously described (*32*). More than 2000 individuals were enrolled throughout the duration of this study, and the mortality rate for the study participants was between 15 and 20%. Consent was given to conduct an autopsy for 103 cases, and these autopsies were performed between 2 and 14 hours after death.

### Selection of samples from autopsy study

For this study, the same human samples previously used by Joice *et al*. (*6*) for assessment of sites of gametocyte enrichment were analyzed. In summary, out of 103 samples, samples from HIV-positive individuals and individuals of unknown HIV status were excluded (27% of enrollees). For the remaining samples, only those for which all nine tissues were available (that is, spleen, heart, brain, lung, liver, BM, gut, kidney, and subcutaneous fat) were assessed. Further filters included a postmortem time before fixation greater than 12 hours (resulting in poor structural preservation of tissue samples) and availability of BM formalin-fixed, paraffin-embedded blocks. This resulted in the selection of a subset of 36 samples. Six were excluded because of lack of CD31 labeling by IHC in the BM sections. From the remaining 30 samples, 22 specimens were selected on the basis of the presence of at least two *Plasmodium* lactate dehydrogenase–labeled parasites per 50 high-power fields in the CD31 analysis.

### Optimization and controls for histological assays in human tissues

To determine antibody specificity for the parasite stages of interest, we generated formalin-fixed and paraffin-embedded mock tissue samples containing cultured RBCs infected with asexual and sexual *P. falciparum* parasite stages. Briefly, 100 μl of packed RBCs, derived either from asexual cultures or from individual time points of gametocyte development time courses, were mixed with thrombin 2 and fetal bovine plasma, wrapped in lens paper, and fixed with formalin. Cell clots were then placed into a paraffin wax block with a composite of surgical tissue specimens obtained from the Brigham and Women’s Hospital (Boston) for BMs that were analyzed in the autopsy study and sliced at a thickness of 3 μm. Using these samples, parasite antibodies and host antibodies were titrated for specificity and against host tissue background labeling.

### IHC assays of human autopsy material

Formalin-fixed and paraffin-embedded tissues and control blocks were cut into 3-μm sections and mounted on slides. Sections were dried overnight at 37°C and then processed through deparaffinization in xylene and subsequently hydrated through a series of graded ethanol (100 to 50%), finishing in water. Antigen retrieval was performed by incubating slides at approximately 95°C in a steamer for 30 min using 1× Antigen Retrieval Reagent Universal (R&D Systems). Following antigen retrieval, slides were blocked using a universal blocking buffer (Thermo Fisher Scientific) for 20 min, followed by 10 min each of avidin- and biotin-blocking buffers (Invitrogen) to block endogenous biotin and avidin, respectively. Antibody incubation [mouse anti-KAHRP (Knob-associated histidine rich protein) combined with rabbit anti-CD31] was then performed overnight at 4°C diluted in blocking buffer (1:100, 1:100, and 1:20 dilutions, respectively, in universal blocking buffer). Wash steps in between each step thereafter were performed with tris-buffered saline + 5% Tween for 3 × 5 min. A secondary goat anti-rabbit horseradish peroxidase–conjugated and a biotin conjugate of the F(ab′)2 fragment of goat anti-mouse immunoglobulin G (IgG) (H + L) antibodies were used (Invitrogen) (diluted to 1:500 in universal blocking buffer), followed by streptavidin conjugated to alkaline phosphatase (Thermo Fisher Scientific) (diluted 1:3000 in universal blocking buffer). For the development of signal, a Fast Red 4 TR/Naphthol AS-MX substrate reagent (Sigma-Aldrich) was applied. Slides were subsequently rinsed in water and counterstained in Mayer’s hematoxylin and mounted in aqueous mounting medium. Slides were blinded to patient identification (ID), and parasites were counted in 100 consecutive high power fields, starting in the upper left corner of each section.

### NanoString expression arrays of human autopsy material

#### RNA extraction

RNA from patient tissue samples stored in RNA*later* was extracted using the Qiagen RNeasy Plus Mini kit (Qiagen). Briefly, tissue samples were homogenized in the Qiagen RLT Lysis Buffer (Qiagen) and processed using the Polytron Homogenizer (Kinematica). The homogenized lysate was passed through genomic DNA eliminator columns (Qiagen), subsequently applied to RNeasy spin columns, followed by several washes according to the manufacturer’s instructions and eluted in nuclease-free water.

#### NanoString analysis

The nCounter custom code set was designed for *P. falciparum* that included differentially expressed genes to distinguish specific stages including asexual parasites (circulating rings and sequestrating trophozoites/schizonts) and sexual stages (gametocyte rings and immature and mature gametocytes) as defined from our previous transcriptional network analysis study (*33*). In total, there are 161 genes representing asexual circulating stages, 147 genes representing asexual sequestering stages, 26 genes representing gametocyte rings, 27 immature gametocytes, and 29 mature gametocyte genes. The remaining set was not annotated for any of these stages. A total of 456 genes were included in this custom probe set including housekeeping genes. For NanoString analysis of tissue samples, 500 ng of purified total RNA was used as input. Briefly, RNA from each sample was allowed to hybridize with reporter and capture probe set at 65°C for 20 hours according to the nCounter gene expression assay protocol (NanoString Technologies). After several washes in the preparation, RNA-probe complexes were immobilized to nCounter cartridge followed by scanning in the nCounter Digital Analyzer. Data were normalized on the basis of background subtraction, and expression of housekeeping genes using nSolver Analysis software (NanoString Technologies) according to guidelines and statistical analysis was performed. The normalized data were included in table S1.

### Animals and ethics statement

Animal studies were carried out under the approval of the Animal Research Ethics Committee of the Canton of Bern, Switzerland (permit numbers 91/11, 81/11, and BE60/17), the University of Bern Animal Care and Use Committee, and the UK Home Office (permit number 60/4443). For all studies, Balb/c, C57BL/6, UBC-GFP C57BL/6, Flk-GFP C57BL/6, and Lys-GFP C57BL/6 females 5 to 12 weeks of age, weighing 25 to 30 g at the time of infection were used. Mice were bred in the central animal facility of the University of Bern or supplied by Harlan Laboratories or Envigo Laboratories. Blood feeding to mosquitoes was performed under Ketasol/Dorbene anesthesia. Luminescence imaging was performed under isofluorane anesthesia, and intravital imaging was performed under xylazine/Ketasol/isofluorane anesthesia, and all efforts were made to minimize suffering.

### *P. berghei* lines used

Various *P. berghei* ANKA lines were used to infect mice. Lines used in this study included Pb mCherry_Hsp70_, constitutively expressing mCherry in the parasite cytosol (*34*); a transgenic line expressing NanoLuc luciferase and mCherry under constitutive promoters (Pb mCherry_Hsp70_NLuc_ef1α_) (*35*); a transgenic line expressing firefly luciferase and mCherry under constitutive promoters Pb mCherry_Hsp70_FLuc_ef1α_ (*36*); and the nonsequestering *P. berghei* line PbΔSBP1-GFP-mCherry_Hsp70_FLuc_ef1α_ (*9*). In addition, *P. berghei* lines G623 (820) and G629 (GNPm9), with CFP expression under the PBANKA_101870 promoter, and lines G488 (820), G458 (GNPm9), and G455(HP), with CFP expression under the Hsp70 promoter were used to investigate differences in parasite localization among previously published gametocyte-producing and gametocyte-nonproducing lines (*15*). Lines G629 (GNPm9) and G455(HP) were used for normalization purposes. Subclones of the above lines were generated by detached cell injection, as previously described (*37*).

### Induction of gametocytemia

To enhance parasitemia and simultaneously induce gametocytemia early after infection as previously published (*8*), erythropoiesis was stimulated by the intraperitoneal injection of 200 μl of a solution of phenylhydrazine in saline (6 mg/ml). Mice were infected (intraperitoneally) with 10^5^ Pb ANKA-mCherry_Hsp70_, G623, G629, G488, or G458 parasites 2 days later. Blood smears were made daily and checked both by fluorescence and Wright staining. Upon reaching 5 to 10% parasitemia, to suppress proliferation of asexual stage parasites, mice received sulfadiazine (10 mg/liter) in their drinking water (from days 3 to 4) after infection onward. Sulfadiazine treatment led to elimination of asexual populations. Because gametocytemia decreases also after 4 or more days of sulfadiazine treatment, IVM, bioluminescence, or collection of infected blood for the purification and passage of gametocytes was routinely performed after 3 to 4 days of treatment (that is, 6 to 7 days after infection).

### Parasite maintenance in mosquitoes

Balb/c mice were treated with phenylhydrazine 3 days before intraperitoneal infection with the different *P. berghei* lines. After 3 days of infection, parasites were spotted on a coverslip, and exflagellation was determined. The infected mice were then used to feed 100 to 150 *Anopheles stephensi* female mosquitoes each. Mice were anesthetized with a combination of Ketasol (125 mg/kg) and Dorbene (12.5 mg/kg) anesthesia and euthanized with CO_2_ after completion of the feed. Afterward, mosquitoes were fed until use with 8% fructose and 0.2% para-aminobenzoic acid. Sporozoites used for mice infection were isolated from the mosquitos’ salivary glands at day 18 after infection.

### Mouse infection with *P. berghei* sporozoites

For bioluminescence imaging of liver stage development, 10^6^ *P. berghei* sporozoites were collected from salivary glands of infected *A. stephensi* mosquitoes and injected intravenously into female Balb/c mice 6 to 12 weeks of age. Liver stage infections were monitored by bioluminescence until egress was detected.

### Mouse infection with *P. berghei* synchronous stages

To generate and purify schizonts and establish synchronous infections, a previously published protocol for schizont transfection was followed (*38*). In summary, two naïve Balb/c mice were injected with 200 μl of a *P. berghei* stabilate. Upon reaching a parasitemia of up to 5% (usually at days 4 to 5 after infection, verified by Wright staining), blood was collected by intracardiac puncture. Complete RPMI1640 culture medium (125 ml) was prepared by adding 25 ml of freshly thawed fetal bovine serum to 100 ml of RPMI1640 culture medium and 200 μl of gentamicin stock solution. Between 15:00 and 18:00, a total of 1.5 to 2 ml of infected blood was collected from each of the two mice by cardiac puncture following euthanasia by CO_2_ inhalation. The blood was immediately added to a 50-ml Falcon tube containing 5 ml of complete media supplemented with 350 μl of heparin stock solution. Infected erythrocytes were then harvested by centrifugation for 8 min at 450*g*, and the supernatant was carefully discarded. The iRBCs were resuspended in 50 ml of complete media and mixed with the 120 ml of RPMI1640 complete media. The total volume was divided equally between two tubes/flasks, each containing around 85 ml. The tubes/flasks were gassed for 90 s at 1.5 to 2 bar pressure with the gas mixture of 5% CO_2_, 5% O_2_, and 90% N_2_. Tubes were closed tightly following gassing and placed immediately at a 37°C shaking/rotating incubator. The parasites were incubated overnight for 16 hours, and viable schizonts were collected between 9:00 and 12:00 of the following morning as follows. First, 500 μl of the schizonts in media was collected and centrifuged for 5 min at 16,000*g*. The supernatant was discarded, and a blood smear was prepared and stained with Wright stain. The smear was verified by light microscopy for contamination and schizont enrichment. Before schizont isolation, a 55% Nycodenz–1× phosphate-buffered saline (PBS) solution (v/v) was prepared in a 50-ml Falcon tube by adding 27.5 ml of Nycodenz solution [138 g of Nycodenz powder dissolved in 500 ml of 5 mM tris/HCl, 3 mM KCl, and 0.3 mM CaNa_2_-EDTA in deionized water (dH_2_O) at pH 7.5] to 22.5 ml of 1× PBS. Schizont cultures (170 ml) were divided into four separate 50-ml Falcon tubes (each containing 25 to 30 ml). Using a 20-ml pipette, 10 ml of Nycodenz-PBS solution was underplayed under the culture suspension in each tube. All tubes were centrifuged for 20 min at 450*g* in a swing-out rotor at room temperature, without brake or initial acceleration. After 20 min, using a Pasteur pipette, the brown-grayish interface of schizonts between the two suspensions was collected from each tube. The isolate (10 μl) was Hoechst-stained, and schizont purity and number per volume were quantified. Aliquots of 6 × 10^6^ schizont were separated into Eppendorf tubes, centrifuged for 5 min at 450*g*, resuspended in 200 μl of 1× PBS, and injected intravenously into naïve UBC-GFP C57BL/6 or WT C57BL/6 mice. Depending on the time when schizont purification was initiated, injection into mice occurred between 10:00 and 13:00. At all consecutive times, mice were kept in a strict 12-hour light cycle to keep parasite synchronicity. Schizont localization in the recipient mice’s bodies (by bioluminescence imaging) or in the BMs (by IVM) was performed at 22 to 24 hours after infection. For isolation of merozoites, rings, and trophozoites, the mice infected with schizonts were regarded as intermediate donor mice and were bled by intracardiac puncture at 4 hours after infection for isolation of rings and at 11 hours after infection for isolation of trophozoites. Upon obtaining 1.5 to 2 ml of synchronously infected blood, a Percoll density gradient was used to separate the corresponding parasite stages. Pure rings or pure trophozoites were resuspended in 200 μl of 1× PBS and intravenously injected into naïve UBC-GFP C57BL/6 or WT C57BL/6 mice. Mice were imaged 2 hours after infection. For merozoite injection, isolated schizonts were mechanically ruptured, and merozoites were briefly incubated in 100 μl of heparin, followed by intravenous injection into naïve UBC-GFP C57BL/6 or WT C57BL/6 mice. Mice were imaged by IVM or bioluminescence immediately following injections.

### Bioluminescence in vivo and organ ex vivo imaging

*Plasmodium* localization was imaged using luciferase-expressing Pb mCherry_Hsp70_FLuc_ef1α_ parasites and gene-deletion mutants PbΔSBP1-GFP-mCherry_Hsp70_FLuc_ef1α_, in a bioluminescence in vivo imaging system (IVIS 100; Caliper Life Sciences—f/stop, 1; no optical filter, or IVIS-Spectrum; Caliper Life Sciences—f/stop, 1; no optical filter) both, in vivo (3-min exposure; 10-min postsubstrate injection; 8 binning; 12.5-cm field view) and ex vivo (30-s exposure; 3-min postsubstrate injection; 8 binning; 10-cm field view). Camera temperature for acquisition was −90°C, and stage temperature during acquisition was 37°C. Bioluminescent images in Balb/c and C57BL/6 mice were obtained at 65 hours after sporozoite intravenous injection, at 22 to 24 hours after infection with synchronous schizonts, at 7 days after intravenous injection of 10^5^ schizonts, and following treatment of mice with phenylhydrazine (2 days before infection) and sulfadiazine (3 days after infection) (treatment described in detail below). Luminescence levels were normalized to schizont levels detected in the brains of control mice. Synchronized infections were established by schizont purification, isolation, and injection as previously described. Mice were anesthetized using 2.5% isofluorane, and 100 ml of the substrate RediJect (30 mg/ml; PerkinElmer, 760504) was injected intraperitoneally. Following whole-body imaging, mice were euthanized and perfused with 1× PBS to remove unbound parasites. Individual organs including the lungs, heart, liver, adipose tissue, BM, kidneys, brain, and spleen were removed from infected mice following perfusion. Acquired data were analyzed using the program Living Image 4.4. Experiments were repeated a minimum of three times, with five mice per repeat.

### Intravital imaging of solid organs

Intravital imaging was performed in 5- to 8-week-old female UBC-GFP C57BL/6 mice, Lys-GFP C57BL/6 mice, Flk1-GFP mice, vascular endothelial-cadherin-GFP mice, WT C57BL/6 mice, and WT Balb/c mice infected with Pb-mCherry_Hsp70_ parasites using synchronous stages (that is, schizonts, merozoites, trophozoites, and gametocytes). Three parasite doses were injected for all experiments involving synchronous infections: an initial inoculum of 10^5^, 10^6^, or 6 × 10^6^. A mixture of anesthetics comprising ketamine (125 mg/kg; Ketasol, Graeub) and xylazine (12.5 mg/kg; Xylasol, Graeub), was prepared and diluted in 1× PBS (1:2:5). Mice were injected intraperitoneally with 100 ml per 20 g of body weight, of the mixed anesthetic, and injections were repeated periodically during imaging. In the case of solid organs, the surgical procedure involved a small incision to expose the spleen, kidney, liver, or adipose tissue. The exposed tissues were glued to a microscope cover glass (VWR 24, 50 mm, no.1) and imaged with a Zeiss LSM5 live scanning module, a Leica SP8-STED confocal microscope, or a Andromeda confocal Spinning Disc microscope with Yanus laser scan head, from Till Photonics. In each microscope, imaging was performed using a Plan Apochromat 63× 1.4 numerical aperture (NA) DIC M27 oil immersion objective (LSM5), an HC PL APO CS2 63× 1.4 NA or 100× oil immersion objective (SP8-STED), or a 60× 1.4 NA oil immersion objective from Olympus. All microscopes were equipped with a temperature chamber to enable controlling the mice’s body temperature following anesthesia during image acquisition. In the Zeiss LSM5 confocal microscope, imaging was performed with 0.1 mW from a 489-nm light from a diode laser to excite GFP, whereas 2 mW from a 561-nm diode pumped solid-state laser was used to excite mCherry. Simultaneous, rather than sequential, excitation was used to ensure high-speed image acquisition. In the Leica SP8-STED microscope, a white laser was used, and wavelengths were defined for the respective spectra of GFP and mCherry. As above, 512 × 512 fields of view were acquired, at speed 4, zoom 10 to 40, and pinhole 1.0. For fast image acquisition, dual fluorescence was simultaneously acquired using an 8000-Hz resonant scanner with 30 times frame averaging. In the Andromeda confocal spinning disc system, GFP was excited with a 488-nm laser, and mCherry was excited with a 561-nm laser. Between 50 and 100 different fields of view were selected in each mouse organ, and images were acquired every 500 ms for 5 min. Experiments were done in triplicate mice for each synchronous parasite stage, each day after infection (that is, 2 hours, days 1 to 7), and the different initial infectious inocula (that is, 10^5^, 10^6^, or 6 × 10^6^). Line scans were positioned at various depths of the vessel, and video-rate imaging was used to record blood and parasite flow in *P. berghei*–infected mice. The recorded movies were assembled and processed using Fiji software (http://fiji.sc/Fiji).

### Intravital imaging of the BM: Calvarium and tibia

For BM IVM, two sites were selected for imaging, namely, the calvarium (skull) and the tibia (leg), to assess potential differences in BM in long bones versus other bone structures. For analysis of the BM in the calvarium, following anesthesia, an incision was made in the skin overlaying the head, within a distance from the eyes to the base of the ears. This resulted in exposure of the skull, which was subsequently mechanically thinned. Measurements in the BM were made between the sagittal suture bifurcation and the intersection of sagittal and coronal sutures. Image postprocessing and analysis were performed using the Fiji software. For IVM of the tibia, mice were prepared as previously published (*39*). In summary, the tibia was exposed by removing the skin on top of the leg, followed by removal of the muscle with a scalpel. The bone was then mechanically thinned. An SP8-STED microscope, or an LSM5 microscope with the features previously described, was used for imaging, and a special mouse holder for the microscope stage was especially designed for this purpose using TopSolid, a CAD/CAM (computer-aided design/computer-aided manufacturing) system. This model was later three dimensionally (3D) printed for use at the SP8-STED microscope.

### Imaging of CFP reporter lines using a SP8-STED microscope

*P. berghei* lines used in this study express CFP, GFP, or mCherry reporters for detection of parasites. When these lines were used in combination with A647 markers to indicate other parameters within the mouse, choice of wavelengths was optimized on the white-laser SP8-STED (fig. S1E). The colors were imaged sequentially, with sequence 1 covering wavelengths 432 to 499 including the excitation maximum of CFP, and measured with a 405-nm laser and a HyD detector for sensitive imaging. Sequence 2 covered wavelengths 500 to 562 including the excitation maximum of GFP and measured using a 488-nm laser and a photomultiplier (PMT) detector. Sequence 3 covered wavelengths 563 to 657 including the excitation maximum of mCherry and measured using a 587-nm laser and a HyD detector. Sequence 4 covered wavelengths 658 to 779, including the maximum of A647, measured using a 653-nm laser and either a HyD or a PMT detector. Imaging ex vivo was done using unidirectional scanning (Galvo scan), a line average of 5, a pinhole of 1.0 AU (Airy units), and varying degrees of zoom. Imaging by IVM was done using the resonance scanner mode, with bidirectional scanning at a rate of 5 frames/s, a pinhole of 3.59 AU, and a line averaging of 5 to 8.

### Assessment of vascular leakage

To visualize the bloodstream, 5 mg of 70-kDa FITC-conjugated dextran (Sigma-Aldrich) and 5 mg of FITC-albumin (Sigma-Aldrich) dissolved in 500 μl of PBS were injected intravenously in the tail vein of an anesthetized mouse. Stacks of 30 images, spaced 1 μm apart, were acquired every minute for volumetric imaging. In addition, time-lapse imaging was acquired as above, that is, between 50 and 100 different fields of view were selected in each mouse organ, and images were acquired every 500 ms for 5 min. FITC was also imaged ex vivo in excised organs. Fluorescence intensity of FITC within and outside the vascular cavities was measured using Fiji.

### Antibody treatment for investigation of the vascular endothelium

To investigate the effects of blocking various molecules present in the vascular endothelium, antibodies used included the following: RB40.34 (rat IgG1, 30 μg per mouse; BD Biosciences, catalog no. 553742), a monoclonal antibody (mAb) against murine P-selectin and 9A9 (rat IgG1, 30 μg per mouse), a mAb against murine E-selectin (provided by K. Ley, La Jolla Institute for Allergy and Immunology) or alternatively a mAb against murine E-selectin (rat IgG1, clone 96419, 30 μg per mouse; R&D Systems no. MAB5751-100). A 1:1 combination of P- and E-selectin antibodies was also used. Likewise, rat mAbs YN1/1.7.4 directed against ICAM-1 (50 μg per mouse, Abcam no. ab25375), rat mAb MK2 directed against VCAM-1 (50 μg per mouse, Abcam no. ab19569), or a 1:1 combination of YN1 and MK2 were used. Antibodies were injected intravenously (E-selectin and P-selectin) or intraperitoneally (all other antibodies) for two consecutive days before injection of purified schizonts/merozoites.

### Transfer of uRBCs to explore basal RBC entry into the BM parenchyma

Three uninfected UBC-GFP mice were bled by cardiac puncture, and RBCs were isolated by centrifugation, after which 10^6^ RBCs (expressing GFP) were resuspended in 200 μl of PBS (per mouse) and injected by tail vein injection. This was repeated in triplicate. No more than 2 hours after UBC-GFP RBC injection, the recipient mice underwent surgery to expose the BM to quantify the localization of the injected RBCs from the donor mice.

### Gametocyte treatment with cytochalasin D

To investigate effects on the capacity of mature gametocytes to transmigrate across the endothelium, donor mice were treated with phenylhydrazine by intraperitoneal injection as previously described and infected 2 days later with 10^6^ *P. berghei* mCherry_Hsp70_FLuc_ef1a_ parasites to produce mixed infections. These donor mice were treated 3 days after infection with sulfadiazine in drinking water (as previously described). Two days later, gametocytes were isolated and further purified by Nycodenz centrifugation (under the same conditions as described for schizont cultures). Purified gametocytes were incubated in 200 nM cytochalasin D (Sigma-Aldrich, catalog no. C8273) in complete RPMI media at 37°C. Four to 6 hours after incubation, parasites were washed 3× in PBS, and 10^6^ parasites were resuspended in 200 μl of PBS (per mouse) and injected intravenously into each one of four recipient Flk1-GFP mice. This experiment was repeated in triplicate. Following injection into the recipient mice, parameters were quantified as follows: (i) total gametocyte numbers (all mice) and (ii) distance from the vasculature within no more than 2 hours after infection (two mice per replicate). Distance was measured to determine whether transmigration still occurred and whether mobility away from the vasculature (and within the parenchyma) was still possible. Measurements of both parameters were performed using either LivingImage 4.4 software for bioluminescence or Fiji for fluorescence-based quantifications.

### Mouse treatment with sildenafil citrate for investigating gametocyte distribution

To investigate the effects of sildenafil citrate on gametocyte deformability in vivo, Balb/c or C57BL/6 mice were pretreated with phenylhydrazine as previously described and infected with 10^6^ *P. berghei* mCherry_Hsp70_FLuc_ef1a_-synchronized schizonts. At day 3 after infection, control mice were treated with sulfadiazine in drinking water, whereas experimental mice were given, in addition to sulfadiazine, daily intraperitoneal injections of sildenafil citrate (European Pharmacopoeia Reference Standard, code: Y0001578, ID: 00G592) for 3 days. Sildenafil citrate (40 mg/kg) was given to various mouse groups, and bioluminescence in the spleen and BM areas was measured daily over the 3 days following treatment using an IVIS-Spectrum (Caliper) system.

### Preparation of mouse organs for immunofluorescence assays

Following IVM, organs were collected for immunofluorescence assays. All organs, including the brain, lungs, heart, adipose tissue, spleen, liver, kidneys, gut, and BM, were poured into a 50-ml Falcon tube containing 1× PBS [137 mM NaCl (Sigma-Aldrich, S9888, 1 kg), 2.7 mM KCl (Fluka Chemie AG P9541, 1 kg), 10 mM Na_2_HPO_4_ (Sigma-Aldrich, S5136, 500 g), 1.8 mM KH_2_PO_4_ (Sigma-Aldrich, P5655), pH 7.4], which was placed on ice or 4°C during further preparations. Initially, each organ was placed on a petri dish and cut into 2-mm slices. The slices of each organ were fixed in 15-ml Falcon tubes containing 2% PFA in PBS, in at least 20× the volume of the tissue (calculated by volume displacement). The Falcon tubes were placed in a rocker for 4 hours. After 4 hours, the PFA solution was removed and replaced with 1× PBS containing sodium azide (NaN_3_) at 20× the tissue volume. Tubes were again placed in a rocker for 30 min. After 30 min, slices were washed three times in cold 1× PBS. After the third wash, 30% sucrose containing NaN_3_ was added to all tissues, covering 20× the volume of the tissues, and organs were then stored overnight at 4°C. The next day, a piece of Styrofoam was placed in a shallow metal container, and dry ice was placed inside this container. 2-Methyl butane was poured over the dry ice, creating a layer of liquid at the bottom of the container, until the depth filled was 1 cm. Before freezing, the 30% sucrose solution was poured away, and the tissue sections were washed twice with 1× PBS. Cryomolds (5 × 3 cm) were used. The cryomolds were then filled with OCT (optimal cutting temperature compound) solution and either transferred immediately to a −80°C freezer or poured into the 2-methyl butane for 5 min. Once frozen, the OCT changes from transparent liquid to solid opaque, at which point the frozen blocks of tissues were placed on foil and kept at −80°C until use. Tissues were then processed for sectioning in 5- to 50-μm slices using a Cryotome cryostat, and slices were placed on glass slides at the point of cutting. The slides were placed at −20°C until use for immunofluorescence assay (IFA).

### Immunofluorescence assays of mouse organs

For each cryostat section, tissues were fixed for 3 min by applying 400 μl of 4% PFA and/or 70% ethanol onto the section, thinly spreading the solution within the entire area of the slide. Tissue sections were then washed twice with 1× PBS at room temperature. Permeabilization solution (200 μl; 1× PBS/0.2% Triton X-100) was spread over the tissue, and slides were incubated in a humid chamber for 10 min. Slides were then washed 3× for 5 min each time, with 1× PBS at room temperature. Slides were blocked using 200 μl of 10% goat serum and incubated in a humid chamber for 1 hour, followed by three washes for 5 min each, with 1× PBS. After blocking, slides were incubated with the diluted primary antibodies overnight at 4°C in a humid chamber. For fluorescent labeling, slides were then incubated with secondary antibodies for 45 min in a humid chamber and then washed 3× with 1× PBS at room temperature. After addition of the secondary antibodies, 200 μl of 4′,6-diamidino-2-phenylindole stain (5 mg/ml) in 1× PBS (1:2000) was poured over the slides and incubated for 2 to 3 min in a humid chamber. The slides were then washed for 5 min in 1× PBS. After allowing the slides to dry slightly (without allowing the tissues to dry out completely), a small drop of VECTASHIELD mounting agent was added, and a 1-mm coverslip was overlayed and pressed gently, ensuring that air bubbles were fully removed. The slides were then sealed with nail polish.

The primary antibodies used were rat monoclonal anti–ICAM-1 (Abcam, catalog no. ab119871) (used at a dilution of 1:100 in 10% goat serum/PBS), rabbit polyclonal anti-CD31 (Abcam, catalog no. ab28364) (used at a dilution of 1:20 in 10% goat serum/PBS), rabbit polyclonal anti-CD71 (used at a dilution of 1:200 in 10% goat serum/PBS), and rabbit polyclonal anti-CD163 (Santa Cruz Biotechnology, catalog no. sc-33560) (used at a dilution of 1:100 in 10% goat serum/PBS). Mouse/human anti-CD44 antibodies conjugated to A647 (BioLegend, catalog no. 103017) were used at a dilution of 1:100 in 10% goat serum/PBS. As secondary antibodies, species-specific Alexa Fluor Cy5 were used at a final concentration of 0.2 μg/ml. Anti-rabbit IgG-Cy5 was used at a dilution of 1:250 and anti-rat IgG-Cy5 was used at a dilution of 1:50 in 10% goat serum/PBS.

### Live imaging of iRBCs in vitro

For determination of parameters for initial machine-learning training settings for staging parasites, time lapse imaging of synchronous stages was performed, using a previously published method (*40*). In summary, 2 to 5 μl of blood were obtained by tail vein puncture, from UBC-GFP transgenic mice infected with synchronous rings, trophozoites, schizonts, or gametocytes of different ages. These were resuspended in 200 μl of 1× PBS and washed by centrifugation at 450*g* for 45 s. The iRBCs were washed two further times and finally resuspended in 500 μl of 1× PBS. Glass-bottom dishes (35 μm; MaTeck) were coated with concanavalin A (0.5 mg/ml) (Sigma-Aldrich) dissolved in dH_2_O. Concanavalin A was added to the glass surface, and the dish was placed in a 37°C incubator for 30 min. After 30 min, it was washed off using 1× PBS, and 250 μl of resuspended iRBCs was overlayed over the glass surface and allowed to settle for 30 min at 37°C. Thereafter, nonbound cells were washed off using PBS, and 2 ml of prewarmed, phenol red–free culture medium was added to the dish. The cells were imaged within a 37°C incubation chamber of an LSM5 confocal microscope (specifics described above). A 63×/1.4 NA oil immersion objective was used, and Zeiss Zen software was used for image acquisition. Cells were viewed using 488-nm (GFP) or 559-nm (mCherry) laser lines simultaneously to detect the RBCs of the UBC-GFP mice and *P. berghei* parasites, respectively. In addition to fluorescence intensity and overall shape, nuclear staining was used to differentiate further the various stages. For this case, Hoechst was used as a nuclear dye and imaged simultaneously using an argon laser to detect the 405-nm wavelength.

### Parasite substaging

For the staging of parasites, we used CellProfiler and CellProfiler Analyst. We partitioned the cells into a training set and a testing set. The bright-field and dark-field features of the training set were used to train the ensemble. Once the ensemble was trained, we evaluated its predictive power on the testing set. To demonstrate the generalizability of this approach and to obtain error bars for our results, the procedure was 10-fold cross-validated. To prevent overfitting the data, the stopping criterion of the training was determined via fivefold internal cross-validation. In addition, we analyzed which features have the most significant contributions for the prediction of parasite stages by “leave one out” cross-validation. The most important features were fluorescence intensity, area, shape, and nuclear staining of the parasites. For the final substaging, manual classifications were always performed for every parasite counted and included in the analysis and cross-checked with the results obtained from the automatic analysis.

### Manual tracking and data analysis

#### Track projections

Gametocyte tracks over time derived from intravital movies were analyzed using the “Cell tracking” and “MTrackJ” ImageJ plugins and ImarisTrack. A total of 500 different gametocytes were tracked for up to 1 min or the duration of their presence in the field of recording.

#### Velocity

Speed of motion was calculated as the sum of distances between track locations, measured using Volocity 6.3 software (PerkinElmer), and divided by track duration. The presence of parasites per movie lasted for varying amounts of time depending on the tissue and extravascular or intravascular presence. For normalization purposes, all calculations were based on 1 to 20 measurements acquired in 500-ms intervals.

#### Calculation of time spent in contact with vessels (lag time)

To calculate the lag time, or the time during which gametocytes made contact with and arrested, videos acquired over a 5-min time lapse were used. Lag time was identified from the calculation of average speed through time (total arrest being equal to zero), as well as average gametocyte speed in relation to the speed of neighboring circulating RBCs or parasites. Lag was considered a decrease in speed by 50% or more compared to total cell and gametocyte speed.

#### Calculation of overall proximity to vessels

In Flk1-GFP mice, the vasculature is GFP-tagged. In UBC-GFP mice, the vascular wall in various organs is visible because of the accumulation of GFP. In addition, in C57BL/6 mice, the vasculature was labeled by injection of FITC-conjugated dextran. Proximity of gametocytes to the vessel wall, as well as number of transmigrations, was calculated using the Volocity 6.3 software (PerkinElmer) or Fiji.

#### Leakage quantification

The images obtained from intravital images of mice injected with FITC-labeled dextran were converted to a false-color scale (rainbow color) based on dextran fluorescent intensity using ImageJ. Then, the blood vessel area and interstitial space were manually circumscribed, and the MFIs of each area were measured every 500 ms. For determination of individual leakage caused by single parasites, a region of interest was generated, and fluorescence changes (MFI) over a 5-min interval were quantified.

#### Statistical analysis

Data were displayed in box plots or histograms generated using PRISM 6.0 software. Means, medians, interquartile ranges, and SDs were calculated from three to five independent experiments performed in triplicate, using STATA 13.0 software. The *P* values were calculated using Student’s *t* test or analysis of variance (ANOVA) in STATA 13.0. Significance between samples is indicated with asterisks as follows: *P < 0.05; **P < 0.01; ***P < 0.001; n.s., not significant.

## Supplementary Material

http://advances.sciencemag.org/cgi/content/full/4/5/eaat3775/DC1

## SUPPLEMENTARY MATERIALS

Supplementary material for this article is available at http://advances.sciencemag.org/cgi/content/full/4/5/eaat3775/DC1 fig. S1. Parasite localization in mixed stage infections and gametocyte sublocalization to BM subcompartments. fig. S2. Parasite clearance, homing, and vascular leakage in BM. fig. S3. Dynamics of vascular leakage during infection. fig. S4. Leakage induced by transmigration and sequestration. fig. S5. Mature gametocyte distribution and mobility. movie S1. Intravital imaging of BM in a Balb/c mouse infected with *P. berghei* ANKA mCherry_Hsp70_ and intravenously injected with 70-kDa FITC-labeled dextran 24 hours after infection. movie S2. Single *P. berghei *mCherry gametocyte moving against the blood flow in a C57BL/6 mouse intravenously injected with FITC-dextran (at 24 hours after infection). movie S3. Compiled movie set of transmigrating gametocyte events. movie S4. Compiled movie sets of gametocyte mobility in UBC-GFP mice. movie S5. Multiple circulating and static purified gametocytes in the sinusoids (S), parenchyma (P), and vasculature (IV) of a UBC-GFP mouse. movie S6. Fast circulation of mature gametocytes within the BM vasculature of a UBC-GFP C57BL/6 mouse. movie S7. Fast circulation of mature gametocytes within the spleen vasculature of a UBC-GFP transgenic mouse. movie S8. Multiple circulating and static purified gametocytes in the sinusoids (S), parenchyma (P), and vasculature (IV) of a UBC-GFP mouse. movie S9. Multiple circulating and static purified gametocytes in the sinusoids (S), parenchyma (P), and vasculature (IV) of a UBC-GFP mouse. movie S10. Multiple circulating and static purified gametocytes in the sinusoids (S), parenchyma (P), and vasculature (IV) of a UBC-GFP mouse. movie S11. Multiple circulating and static purified gametocytes in the sinusoids (S), parenchyma (P), and vasculature (IV) of a UBC-GFP mouse. movie S12. Multiple fast-circulating gametocytes in a vascular tree of the spleen of a UBC-GFP mouse. movie S13. Leukocyte motility, crawling adhesion, diapedesis (image center and top), and accumulation at the vessel wall (image bottom) of a Lys-GFP control mouse. table S1. This table contains the normalized expression values across all the 456 genes included in the NanoString expression array.
