# Supplementary material for: Plasmodium gametocytes display homing and vascular transmigration in the host bone marrow
Source: Sci Adv. 2018 May 23;4(5):eaat3775. doi: 10.1126/sciadv.aat3775 (PMC5966192; doi:10.1126/sciadv.aat3775)
Supplement: http://advances.sciencemag.org/cgi/content/full/4/5/eaat3775/DC1 [file supp_4_5_eaat3775__index.html]

Science Advances | Science Advances

## Supplementary Materials

**This PDF file includes:**

- fig. S1. Parasite localization in mixed stage infections and gametocyte sublocalization to BM subcompartments.
- fig. S2. Parasite clearance, homing, and vascular leakage in BM.
- fig. S3. Dynamics of vascular leakage during infection.
- fig. S4. Leakage induced by transmigration and sequestration.
- fig. S5. Mature gametocyte distribution and mobility.
- Legends for movies S1 to S13
- Legend for table S1

Download PDF

**Other Supplementary Material for this manuscript includes the following:**

- movie S1 (.avi format). Intravital imaging of BM in a Balb/c mouse infected with *P. berghei* ANKA mCherryHsp70 and intravenously injected with 70-kDa FITC-labeled dextran 24 hours after infection.
- movie S2 (.avi format). Single *P. berghei* mCherry gametocyte moving against the blood flow in a C57BL/6 mouse intravenously injected with FITC-dextran (at 24 hours after infection).
- movie S3 (.avi format). Compiled movie set of transmigrating gametocyte events.
- movie S4 (.avi format). Compiled movie sets of gametocyte mobility in UBC-GFP mice.
- movie S5 (.avi format). Multiple circulating and static purified gametocytes in the sinusoids (S), parenchyma (P), and vasculature (IV) of a UBC-GFP mouse.
- movie S6 (.avi format). Fast circulation of mature gametocytes within the BM vasculature of a UBC-GFP C57BL/6 mouse.
- movie S7 (.avi format). Fast circulation of mature gametocytes within the spleen vasculature of a UBC-GFP transgenic mouse.
- movie S8 (.avi format). Multiple circulating and static purified gametocytes in the sinusoids (S), parenchyma (P), and vasculature (IV) of a UBC-GFP mouse.
- movie S9 (.avi format). Multiple circulating and static purified gametocytes in the sinusoids (S), parenchyma (P), and vasculature (IV) of a UBC-GFP mouse.
- movie S10 (.avi format). Multiple circulating and static purified gametocytes in the sinusoids (S), parenchyma (P), and vasculature (IV) of a UBC-GFP mouse.
- movie S11 (.avi format). Multiple circulating and static purified gametocytes in the sinusoids (S), parenchyma (P), and vasculature (IV) of a UBC-GFP mouse.
- movie S12 (.avi format). Multiple fast-circulating gametocytes in a vascular tree of the spleen of a UBC-GFP mouse.
- movie S13 (.mov format). Leukocyte motility, crawling adhesion, diapedesis (image center and top), and accumulation at the vessel wall (image bottom) of a Lys-GFP control mouse.
- table S1 (Microsoft Excel format). This table contains the normalized expression values across all the 456 genes included in the NanoString expression array.

**Files in this Data Supplement:**

- Adobe PDF - aat3775\_SM.pdf
