# Supplementary material for: Plasmodium gametocytes display homing and vascular transmigration in the host bone marrow
Source: Sci Adv. 2018 May 23;4(5):eaat3775. doi: 10.1126/sciadv.aat3775 (PMC5966192; doi:10.1126/sciadv.aat3775)
Supplement: http://advances.sciencemag.org/cgi/content/full/4/5/eaat3775/DC1 [file aat3775_SM.pdf]

Science Advances

AAAS

advances.sciencemag.org/cgi/content/full/4/5/eaat3775/DC1

Supplementary Materials for

Plasmodium gametocytes display homing and vascular transmigration in the host bone marrow

Mariana De Niz, Elamaran Meibalan, Pedro Mejia, Siyuan Ma, Nicolas M. B. Brancucci, Carolina Agop-Nersesian, Rebecca Mandt, Priscilla Ngotho, Katie R. Hughes, Andrew P. Waters, Curtis Huttenhower, James R. Mitchell, Roberta Martinelli, Friedrich Frischknecht, Karl B. Seydel, Terrie Taylor, Danny Milner, Volker T. Heussler, Matthias Marti

Published 23 May 2018, *Sci. Adv.* **4**, eaat3775 (2018)
DOI: 10.1126/sciadv.aat3775

The PDF file includes:

- fig. S1. Parasite localization in mixed stage infections and gametocyte sublocalization to BM subcompartments.
- fig. S2. Parasite clearance, homing, and vascular leakage in BM.
- fig. S3. Dynamics of vascular leakage during infection.
- fig. S4. Leakage induced by transmigration and sequestration.
- fig. S5. Mature gametocyte distribution and mobility.
- Legends for movies S1 to S13
- Legend for table S1

Other Supplementary Material for this manuscript includes the following:
(available at advances.sciencemag.org/cgi/content/full/4/5/eaat3775/DC1)

- movie S1 (.avi format). Intravital imaging of BM in a Balb/c mouse infected with *P. berghei* ANKA mCherry$_{Hsp70}$ and intravenously injected with 70-kDa FITC-labeled dextran 24 hours after infection.
- movie S2 (.avi format). Single *P. berghei* mCherry gametocyte moving against the blood flow in a C57BL/6 mouse intravenously injected with FITC-dextran (at 24 hours after infection).
- movie S3 (.avi format). Compiled movie set of transmigrating gametocyte events.
- movie S4 (.avi format). Compiled movie sets of gametocyte mobility in UBC-GFP mice.
- movie S5 (.avi format). Multiple circulating and static purified gametocytes in the sinusoids (S), parenchyma (P), and vasculature (IV) of a UBC-GFP mouse.

- movie S6 (.avi format). Fast circulation of mature gametocytes within the BM vasculature of a UBC-GFP C57BL/6 mouse.
- movie S7 (.avi format). Fast circulation of mature gametocytes within the spleen vasculature of a UBC-GFP transgenic mouse.
- movie S8 (.avi format). Multiple circulating and static purified gametocytes in the sinusoids (S), parenchyma (P), and vasculature (IV) of a UBC-GFP mouse.
- movie S9 (.avi format). Multiple circulating and static purified gametocytes in the sinusoids (S), parenchyma (P), and vasculature (IV) of a UBC-GFP mouse.
- movie S10 (.avi format). Multiple circulating and static purified gametocytes in the sinusoids (S), parenchyma (P), and vasculature (IV) of a UBC-GFP mouse.
- movie S11 (.avi format). Multiple circulating and static purified gametocytes in the sinusoids (S), parenchyma (P), and vasculature (IV) of a UBC-GFP mouse.
- movie S12 (.avi format). Multiple fast-circulating gametocytes in a vascular tree of the spleen of a UBC-GFP mouse.
- movie S13 (.mov format). Leukocyte motility, crawling adhesion, diapedesis (image center and top), and accumulation at the vessel wall (image bottom) of a Lys-GFP control mouse.
- table S1 (Microsoft Excel format). This table contains the normalized expression values across all the 456 genes included in the NanoString expression array.

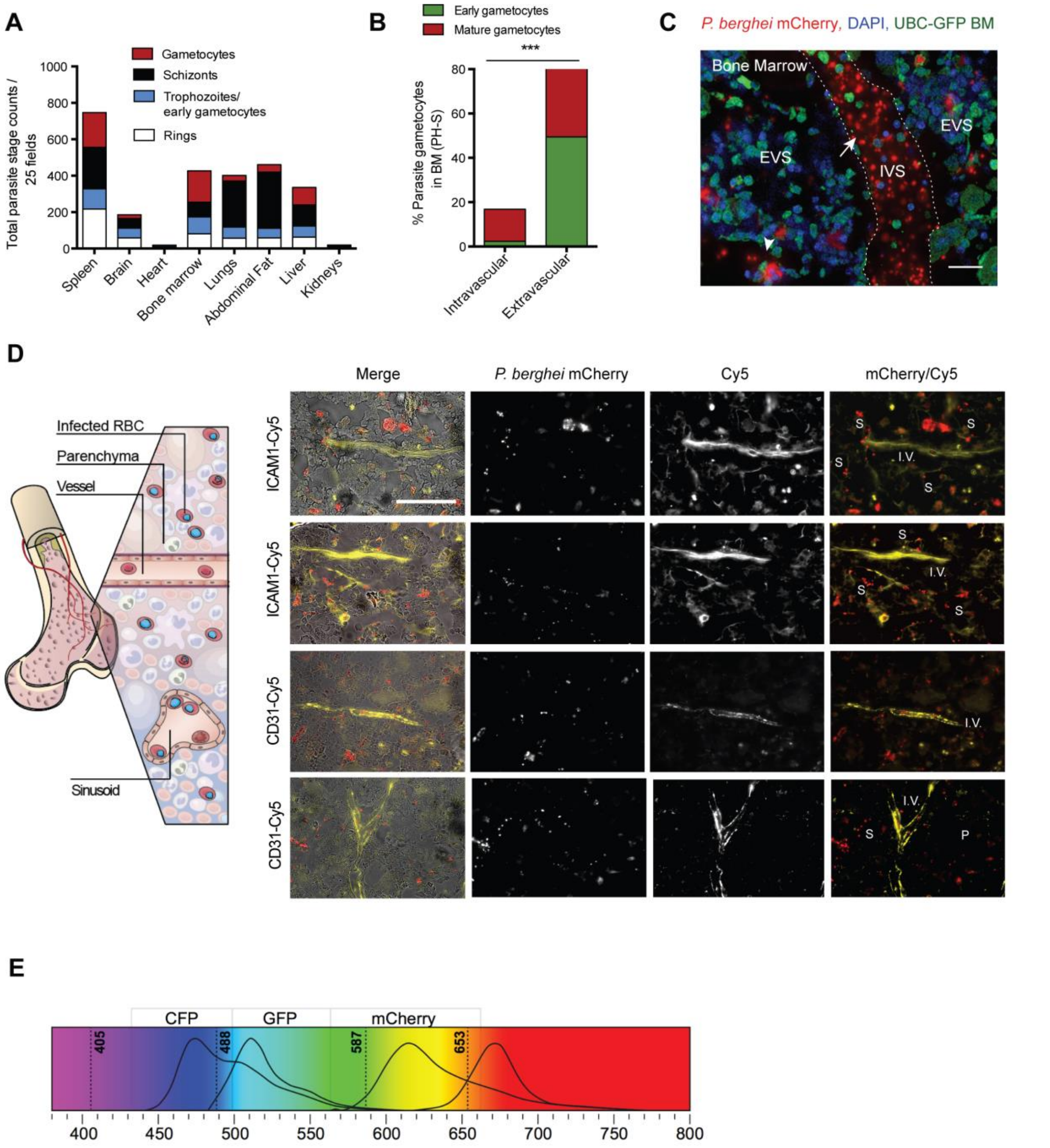

fig. S1. Parasite localization in mixed stage infections and gametocyte sublocalization to BM subcompartments. (**A**) Quantification of total parasite load by stage and organ. *P. berghei* mCherry$_{\mathrm{Hsp70}}$-FLuc$_{\mathrm{ef1}\alpha}$ parasites were analyzed by intravital microscopy and *ex vivo* imaging. Total parasite burden was quantified, and stratified by stage using classifications made by CellProfiler on purified stages. Parasite numbers were lowest in the heart, brain and kidneys and highest in the spleen. The spleen shows highest ring presence, while schizonts are highly enriched in lungs, adipose tissue, liver and spleen. Gametocytes are highly enriched in the spleen, bone marrow and liver. (**B**) Quantification of intravascular and

extravascular localization of mCherry$_{Hsp70}$-FLuc$_{ef1\alpha}$ gametocytes in the bone marrow, based on *ex vivo* imaging and CD31+ labeling in PH-S treated mice. Immature gametocytes are virtually absent in the vasculature. (**C**) Representative images of the *P. berghei* mCherry$_{Hsp70}$-FLuc$_{ef1\alpha}$ distribution in the extravascular (EVS, white arrowhead) and intravascular space (IVS, white arrow) of the bone marrow. (**D**) Schematic showing compartmentalization into vasculature, sinusoids, and parenchyma (left). Representative images showing IFAs from bone marrows of mice infected with *P. berghei* mCherry, and labeled using antibodies against ICAM1 or CD31 (shown in yellow) for definition of intravascular (I.V.), sinusoidal (S) and parenchymal (P) compartments. (**E**) Spectrum of imaging sequences used in the SP8-STED Leica microscope for imaging gametocyte reporter *P. berghei* lines including CFP (early gametocytes or constitutive expression), GFP (mature male gametocytes) and mCherry (mature female gametocytes). Each color was imaged under a separate sequence, using a tunable white laser and a UV laser (see also materials and methods). CFP was imaged within wavelengths 432-499nm using the 405 laser as shown in the diagram. GFP was imaged within wavelengths 499-558nm. mCherry was imaged within wavelengths 558-606nm, and A647 (used for imaging additional reporters of vascular leakage or erythroid precursors, for example) was imaged within wavelengths 606-794nm.

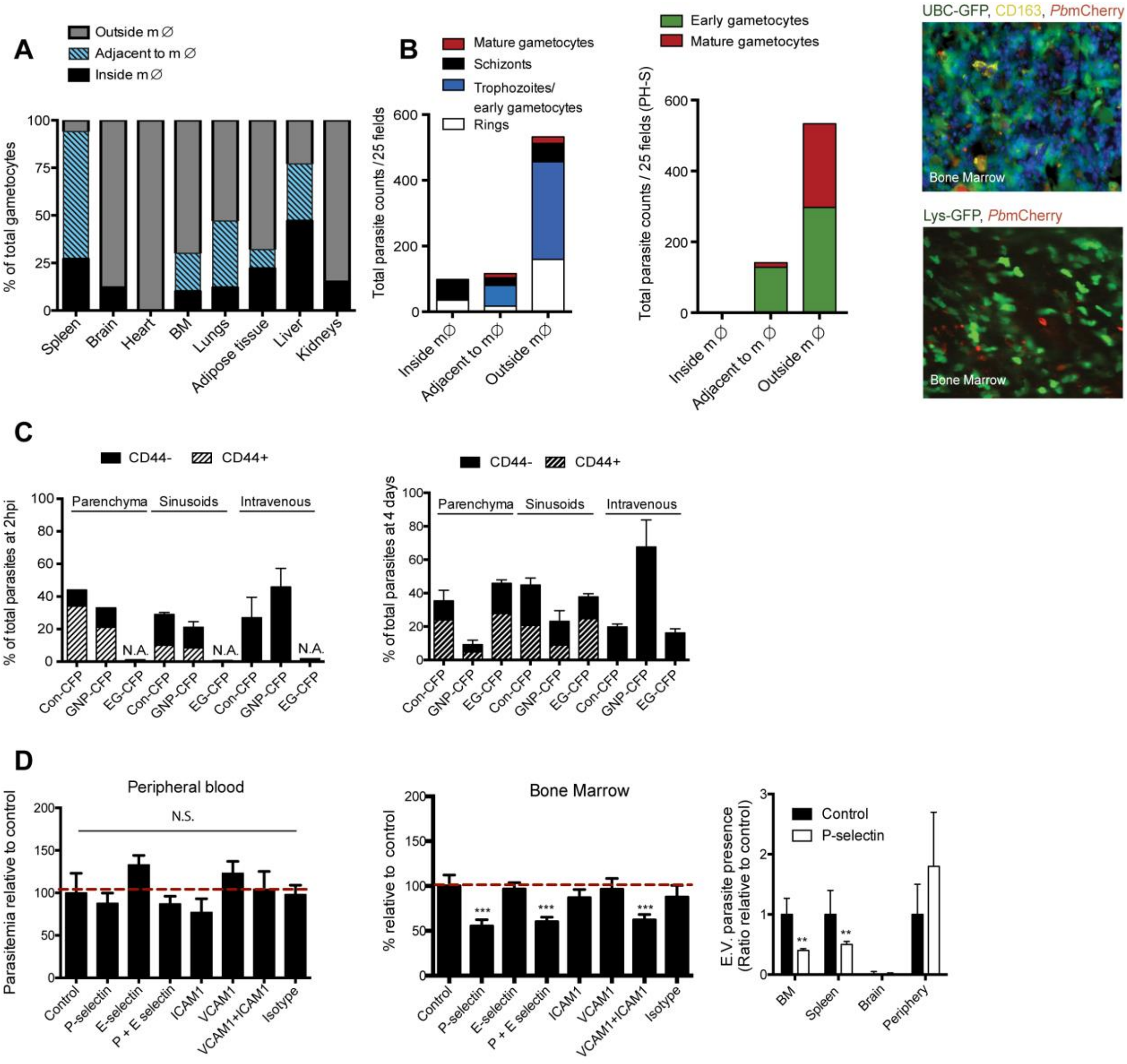

fig. S2. Parasite clearance, homing, and vascular leakage in BM. (**A**) Parasite distribution relative to macrophages. Quantification of mCherry$_{Hsp70}$-FLuc$_{ef1\alpha}$ gametocyte localization across tissues, relative to macrophages, as assessed by IFA quantification of tissues stained for CD163, or localization relative to Lys-GFP^{+} cells in reporter mice. Liver, spleen and adipose tissue show highest levels of phagocytosed parasites. (**B**) Parasite stage distribution relative to macrophages in BM. *P. berghei* mCherry$_{Hsp70}$-FLuc$_{ef1\alpha}$ parasite localization within the BM is quantified relative to macrophages. Rings and schizonts were preferentially found inside macrophages, while mostly rings and trophozoite-sized parasites were found outside macrophages. Representative images showing CD163 labeling (yellow), and the relative localization of *P. berghei* parasites (red) in the BM of UBC-GFP mice (green)(top image). Intravital image of *P. berghei* parasites (red) in the BM of Lys-GFP mice, reporters for the myeloid lineage (green)(bottom image). (**C**) Presence of stage-specific reporter parasites in

CD44+ RBCs following injection of merozoites. At day 4 pi EG-CFP parasite are mostly confined to the parenchyma and sinusoids, yet with some presence in the intravascular space unlike at 24 hpi. Con-CFP parasites show similar distribution to the EG-CFP parasites, except for a higher presence in CD44- cells within the sinusoidal compartments. GNP-CFP parasites are preferentially in the intravenous compartment followed by the sinusoids, with only a minority of parasites in the BM parenchyma. N.A. = Not analyzed, as EG-CFP reporter is not expressed at this time point. (**D**) Inhibition of parasite extravasation using receptor antibodies. Mice were pretreated for 24-48 h with various receptor antibodies (individually or in combination) before injection of $mCherry_{Hsp70}$-$FLuc_{ef1\alpha}$ merozoites, and compared to untreated control mice. Parasite abundance in BM is significantly reduced upon treatment with P-selectin, or combinations of P-selectin and E-selectin or VCAM1 and ICAM1. No differences are observed in the peripheral blood. (**$p<0.01$; ***$p<0.001$. Error bars represent S.D.)

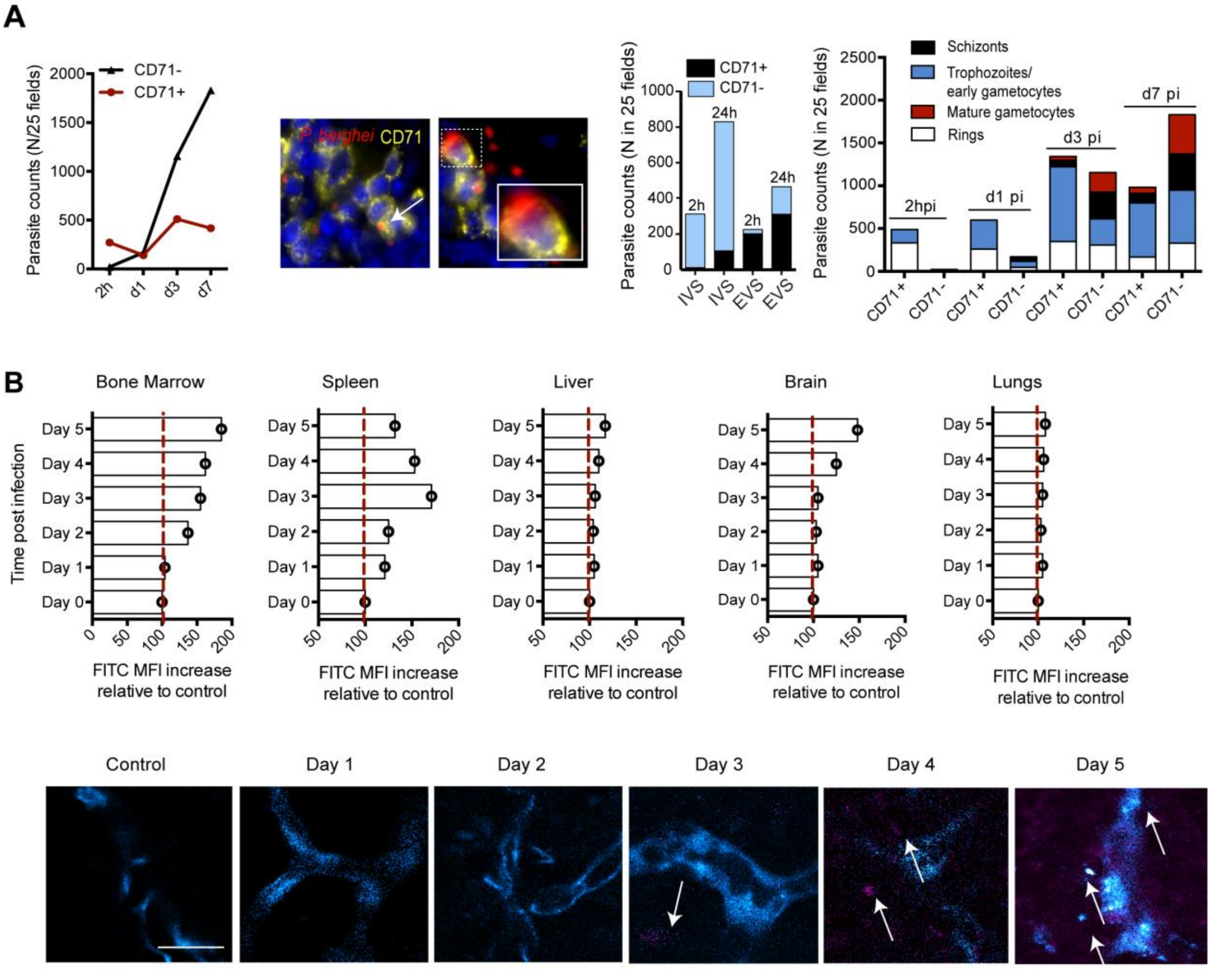

fig. S3. Dynamics of vascular leakage during infection. (**A**) Quantification of mCherry$_{Hsp70}$-FLuc$_{ef1\alpha}$ parasites within CD71$^+$ (erythroid precursors and reticulocytes) and CD71$^-$ cells (mature RBCs) over time (left panel) and in BM compartments (right panels). The majority of parasites in the extravascular niche are in CD71+ cells at 2 hpi and 24 hpi after injection of merozoites (mid right panel). Increasing numbers of schizonts are detected in the BM within and outside CD71+ later during infection (d3 and d7 pi)(right panel). Representative images of parasites within CD71+ cells are shown. (**B**) Vascular leakage across tissues during mouse infection. C57BL/6 mice were infected with 10^6 parasites intravenously and injected with FITC-labeled dextran to measure vascular leakage from d1 to d5 pi. Uninfected mice were used as control. Mean fluorescence intensity (MFI) measurements in mice at different time points pi show progressive increase of vascular leakage (top panel). Representative images from bone marrow are shown in the bottom panel. Scale bar = 50 µm.

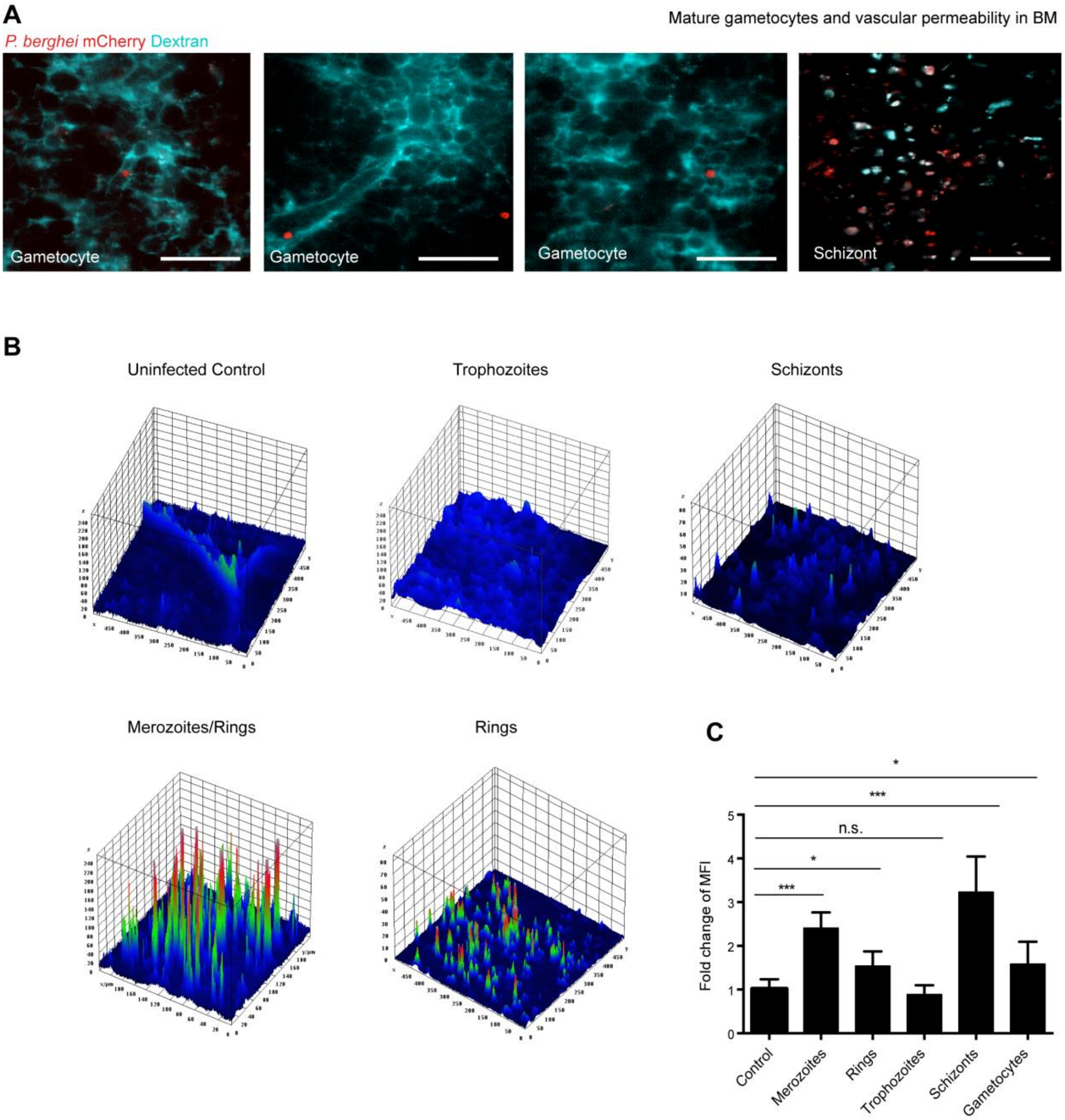

fig. S4. Leakage induced by transmigration and sequestration. (**A-C**) FITC-Dextran injections in mice infected with synchronous parasites. (A) Shows representative FITC-labeled Dextran accumulations in mice infected with purified gametocytes or schizonts, obtained from IVM movies. While gametocytes move against the blood flow, and do not induce significant sustained vascular leakage, schizonts show increased accumulations of FITC, consistent with increased vascular leakage. (B) 3D projections of vascular leakage in control uninfected mice, and ring-, trophozoite-, schizont- and merozoite-infected mice, based on FITC-labeled Dextran vasculature imaged by IVM. Note that schizont sequestration to the vascular endothelium itself may be responsible for the observed increased vascular

permeability in focal points. (C) Mean fluorescence intensity (MFI) of average leakage in 50 different frames through multiple time points in mice infected with synchronous stages (merozoites/rings, rings, trophozoites, schizonts, or gametocytes). *$p < 0.05$; ***$p<0.001$.

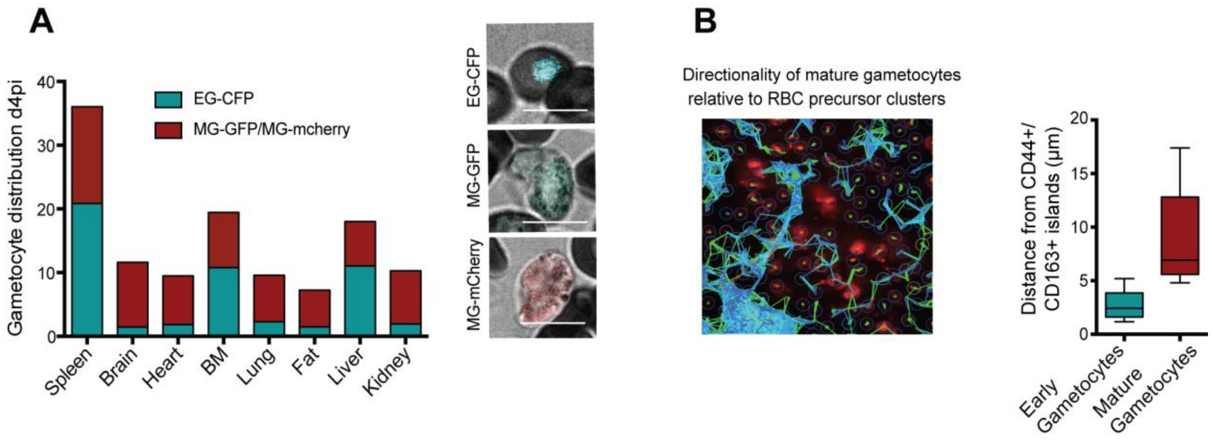

fig. S5. Mature gametocyte distribution and mobility. (**A**) Mature and early gametocyte distribution across organs. Parasites were classified into early (EG-CFP) and mature (MG-GFP or MG-mCherry) and quantified across tissues in mice day 4 pi (left panel). Early gametocytes are greatly enriched in BM, spleen and liver. Right panel: Images show early (CFP, top), and mature male (GFP, middle) or mature female (mCherry, bottom) gametocytes. Scale bars = 20µm. (**B**) Directionality of movement and distribution of gametocytes in the BM. Left panel: Mature gametocytes display random and non-directional mobility in the extravascular niche relative to erythroid cells. Right panel: Average distance of mature (red) and early (blue) gametocytes relative to erythroblast islands (CD44+ or CD163+ cells).

Supplementary movie captions

movie S1. Intravital imaging of BM in a Balb/c mouse infected with *P. berghei* ANKA mCherry$_{Hsp70}$ and intravenously injected with 70-kDa FITC-labeled dextran 24 hours after infection. Rainbow intensity ranges from blue (no leakage) to green (medium permeability) to red (significant vascular leakage) based on fluorescence intensity. Various punctual leakage points can be observed at time 0.00s, which increase in intensity over time. On the first frame, the arrow shows the direction of blood flow. Subsequent arrowheads show increased punctual dextran accumulations, which suggest increased permeability. Scale bar is 20 μm.

movie S2. Single *P. berghei* mCherry gametocyte moving against the blood flow in a C57BL/6 mouse intravenously injected with FITC-dextran (at 24 hours after infection). The direction of blood flow is marked by a solid arrow. The gametocyte is located in the right side of the frame resisting blood flow from 0.0s to 21.50s. Afterwards it is carried with the blood flow, reappearing eventually at 34.5s. Scale bar is 20 μm.

movie S3. Compiled movie set of transmigrating gametocyte events. Movie 1 was acquired in Flk1-GFP transgenic mice (vasculature marked in white). Mice were infected with purified mature gametocytes to observe their migration. At 0.2s the arrowhead points at a circulating gametocyte within the vasculature following until 0.8s, after which crossing occurs (marked by a T), whereby the gametocyte leaves the demarcated vasculature. A similar phenomenon is repeated between 7.2s and 8.8s. Movies 2-5 were acquired in the spleen of UBC-GFP transgenic mice (UBC-GFP constitutively expressed in all tissues, however the delineated vasculature can be clearly visualized). In movie 2 the mature gametocyte is observed behind the field of view where the vessel is visible from 0 to 0.8s. Between 1.4 and 3.4s, the gametocyte crosses the vascular endothelium. Movie 3 shows a long-term crossing gametocyte, which comes in contact with the parenchyma at 8.8s, and continues migrating until 18s. Movie 4 shows two gametocytes sequentially navigating the BM parenchyma from 0.2s to 12.0s. Movie 5 shows another transmigration event lasting 4.0s. Scale bar is 20 μm.

movie S4. Compiled movie sets of gametocyte mobility in UBC-GFP mice. Movie 1 in the BM parenchyma shows an arrowhead following a gametocyte moving from 0.0s to 33.4s Movie 2 shows an arrowhead following a mature gametocyte moving in “U” motion in a phenomenon lasting 19.6s. Movie 3 shows a mature gametocyte initially trapped within the BM parenchyma, and then moving through, within 15.2s. Movie 4 shows a mature gametocyte in the mouse spleen, deforming and moving within an event lasting almost a minute. Movie 5 shows a mature gametocyte in the spleen, changing direction and moving upward within an event lasting 24.4s. Scale bar is 20 μm.

movie S5. Multiple circulating and static purified gametocytes in the sinusoids (S), parenchyma (P), and vasculature (IV) of a UBC-GFP mouse. No transmigrations occur. Scale bar is 20 μm.

movie S6. Fast circulation of mature gametocytes within the BM vasculature of a UBC-GFP C57BL/6 mouse. Scale bar is 20 μm.

movie S7. Fast circulation of mature gametocytes within the spleen vasculature of a UBC-GFP transgenic mouse. Only one parasite is visibly static in the vasculature in the upper right part of the frame. Scale bar is 20 μm.

movie S8. Multiple circulating and static purified gametocytes in the sinusoids (S), parenchyma (P), and vasculature (IV) of a UBC-GFP mouse. No transmigrations occur. Static gametocytes are shown by an arrowhead. Scale bar is 20 μm.

movie S9. Multiple circulating and static purified gametocytes in the sinusoids (S), parenchyma (P), and vasculature (IV) of a UBC-GFP mouse. No transmigrations occur. Static gametocytes are shown by an arrowhead. Scale bar is 20 μm.

movie S10. Multiple circulating and static purified gametocytes in the sinusoids (S), parenchyma (P), and vasculature (IV) of a UBC-GFP mouse. No transmigrations occur. Static gametocytes are shown by an arrowhead. Scale bar is 20 μm.

movie S11. Multiple circulating and static purified gametocytes in the sinusoids (S), parenchyma (P), and vasculature (IV) of a UBC-GFP mouse. No transmigrations occur. Static gametocytes are shown by an arrowhead. Scale bar is 20 μm.

movie S12. Multiple fast-circulating gametocytes in a vascular tree of the spleen of a UBC-GFP mouse. No transmigrations occur. Scale bar is 20 μm.

movie S13. Leukocyte motility, crawling adhesion, diapedesis (image center and top), and accumulation at the vessel wall (image bottom) of a Lys-GFP control mouse. Scale bar is 50 μm.

Supplementary table captions

table S1. This table contains the normalized expression values across all the 456 genes included in the NanoString expression array. Shown are data across 9 patients (PM91, 92, 94, 95, 96, 98, 99, 100, 101) and 3 tissues (bone marrow, brain and heart).
